# Utilizing Raman Spectroscopy as a Tool for Solid- and Solution-Phase Analysis of Metalloorganic Cage Host–Guest Complexes

**DOI:** 10.1021/acs.inorgchem.2c00873

**Published:** 2022-05-05

**Authors:** Helen M. O’Connor, William J. Tipping, Julia Vallejo, Gary S. Nichol, Karen Faulds, Duncan Graham, Euan K. Brechin, Paul J. Lusby

**Affiliations:** ‡ EaStCHEM School of Chemistry, The University of Edinburgh, David Brewster Road, Edinburgh EH9 3FJ, U.K.; § Pure and Applied Chemistry, Technology and Innovation Centre, University of Strathclyde, 99 George Street, Glasgow G1 1RD, U.K.

## Abstract

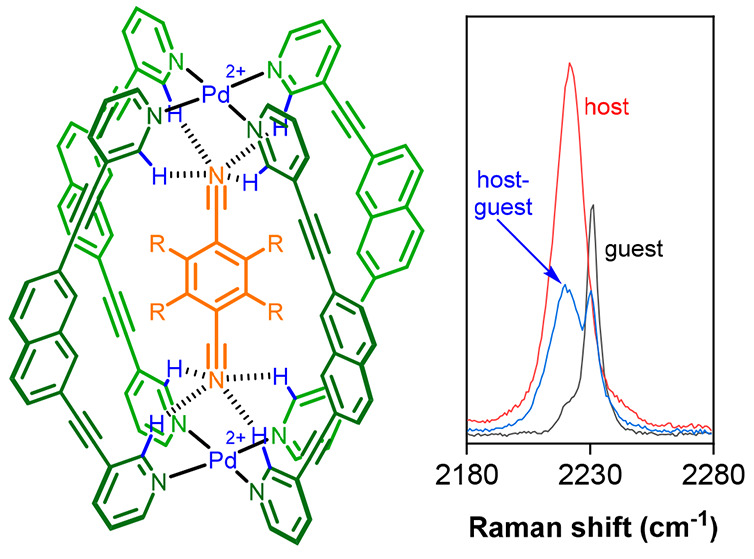

The host–guest chemistry of coordination cages continues to promote significant interest, not least because confinement effects can be exploited for a range of applications, such as drug delivery, sensing, and catalysis. Often a fundamental analysis of noncovalent encapsulation is required to provide the necessary insight into the design of better functional systems. In this paper, we demonstrate the use of various techniques to probe the host–guest chemistry of a novel Pd_2_L_4_ cage, which we show is preorganized to selectively bind dicyanoarene guests with high affinity through hydrogen-bonding and other weak interactions. In addition, we exemplify the use of Raman spectroscopy as a tool for analyzing coordination cages, exploiting alkyne and nitrile reporter functional groups that are contained within the host and guest, respectively.

## Abstract 2

The host−guest chemistry of a novel Pd_2_L_4_ cage has been investigated. This cage shows a strong affinity toward dicyanoarene compounds, with affinities ranging from 10^3^ to 10^4^ M^−1^. NMR spectroscopy and X-ray crystallography reveal that the guests are bound through CH···N hydrogen-bonding and CH···π interactions, with subtle distortions also affecting the binding strength. The use of Raman spectroscopy as a tool for analyzing coordination cages, exploiting alkyne and nitrile reporter groups, is also highlighted.

## Introduction

Analysis of the interactions that govern encapsulation phenomena is crucial for an understanding of the metallosupramolecular host–guest complexes that find application in areas such as catalysis, drug delivery, molecular recognition, and separation.^1−3^ Single-crystal X-ray crystallography is the most definitive tool for demonstrating the formation of a host–guest complex because it allows encapsulation to be directly visualized. It can also provide compelling evidence as to which noncovalent interactions may be key to binding.^4−6^ Solution host–guest studies, using techniques such as ^1^H NMR spectroscopy and spectrophotometric methods, are also key, not least because many of the most important applications occur in this phase. It is especially important to show that encapsulation observed in the solid state persists in solution, where sometimes the competition from a vast excess of even weakly interacting solvent molecules can disrupt the interactions that drive binding.

IR and Raman (RS) spectroscopy have been much less widely used for probing host–guest compounds. However, they are powerful methods for analyzing molecular structure and can be used to monitor the changes in specific vibrational modes of the guest and host upon complexation.^7−15^ They are also advantageous because they can be applied to both solution- and solid-phase (crystalline and noncrystalline) samples. Raman analysis of metalloorganic cages is underexplored; however, there are clear opportunities.^16,17^ Specifically, the alkyne functional group, which is often used as a structurally rigid spacer unit in many coordination assemblies, is a widely used RS handle. This is because its stretching frequency generates intense peaks^18^ and is both highly sensitive to changes in the local environment^19,20^ and direct chemical modification.^21^ It also occurs in a largely silent region of the spectrum, which, in particular, has made it a popular choice for analyzing suitably labeled biomolecules within cells.^18^

**Figure 1:**
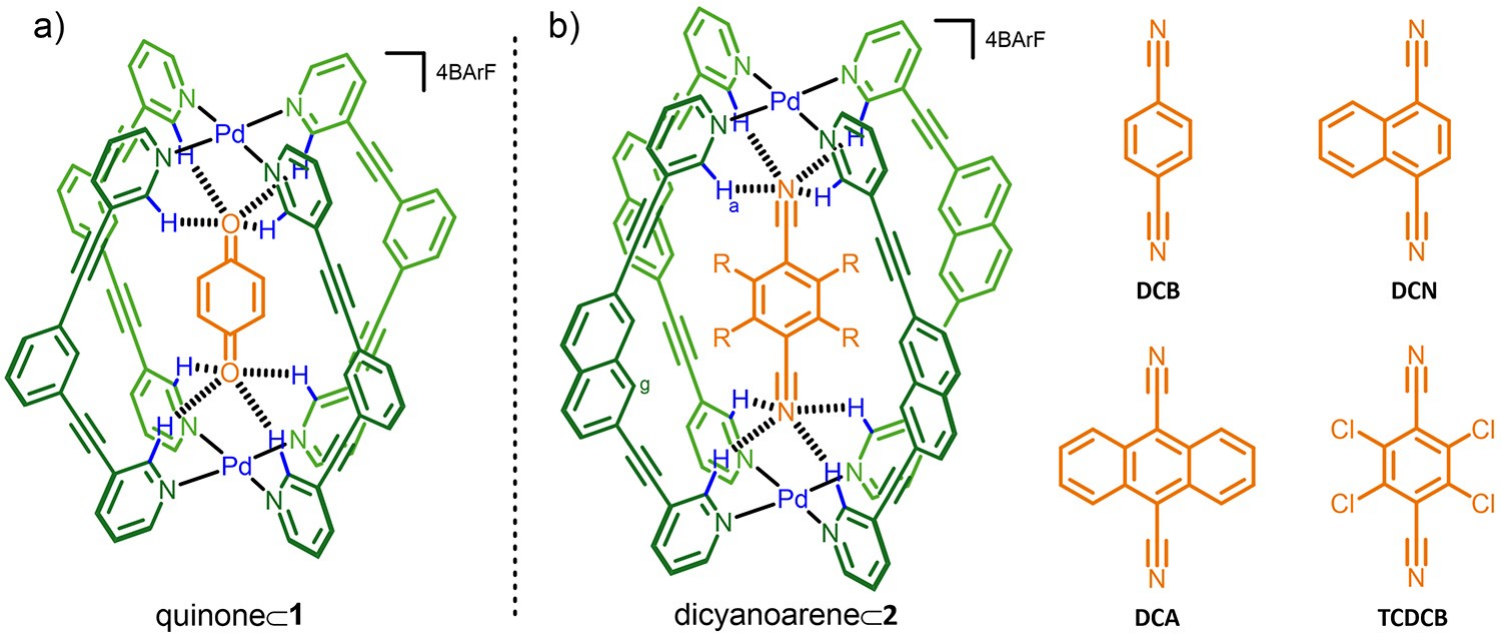
Chemical structures of (a) the previously studied quinone⊂**1** host–guest cage complex and (b) the dicyanoarene⊂**2** inclusion complex alongside the different guests investigated in this current study.

An example of an alkyne-containing metalloorganic cage is compound **1**. We have previously studied the host–guest chemistry of **1** (Figure 1a), showing that it is complementary toward quinones.^22^ This is because the O–O distance of the guest is perfectly matched to form hydrogen-bonding interactions with both pockets of *o*-pyridyl CH H-bond donors (Figure 1a, where H atoms are shown in blue). We have also shown that bound quinones can act as both substrates and cofactors in catalytic investigations, wherein the activity comes from electronic modulation of the encapsulated species.^23,24^ Our most recent catalytic investigations have shown that substrates with a range of H-bond-accepting functional groups can interact with the cage.^25^ In the context of RS, we were particularly interested in guests that contain a nitrile group; this is also a useful spectroscopic handle because, like the alkyne stretch, it occurs in the region of the spectrum (≈2100–2300 cm^–1^) where there are few other signals. We herein describe the use of RS as a complementary tool for probing the binding properties of a Pd_2_L_4_ metalloorganic cage, exploiting a dual-labeled, alkyne–nitrile host–guest system (Figure 1b).

## Results and Discussion

*p*-Dicyanoaryl compounds were an obvious choice of guest because (a) it was hoped that their vertically aligned nitrile groups could simultaneously interact with both pockets of the H-bond donors in a fashion analogous to that of quinone⊂**1** and (b) there are several dicyanoarenes that are commercially available. To accommodate the larger separation between the nitrile N atoms of the dicyanoarene guests compared to the interoxygen distance in a quinone, we targeted cage **2** (Figure 1b), which features a naphthyl spacer instead of the *m*-C_6_H_4_ motif that is used in the parent cage **1**. The ligand for cage **2** was prepared by Sonogashira cross-coupling from commercially available materials.^26^ Combining 2 equiv of this ligand with [(CH_3_CN)_4_Pd](OTf)_2_ in equal amounts of CH_3_CN/CH_2_Cl_2_ led to formation of the expected [Pd_2_L_4_](OTf)_4_ structure, as evidenced by NMR spectroscopy (Figure S2). Anion metathesis with the noncoordinating tetrakis[3,5-bis(trifluoromethyl)phenyl]borate (BArF) gave [Pd_2_L_4_](BArF)_4_ (**2**). We have previously described how the use of BArF counteranions can maximize host–guest interactions with neutral organic molecules.^22^

**Figure 2:**
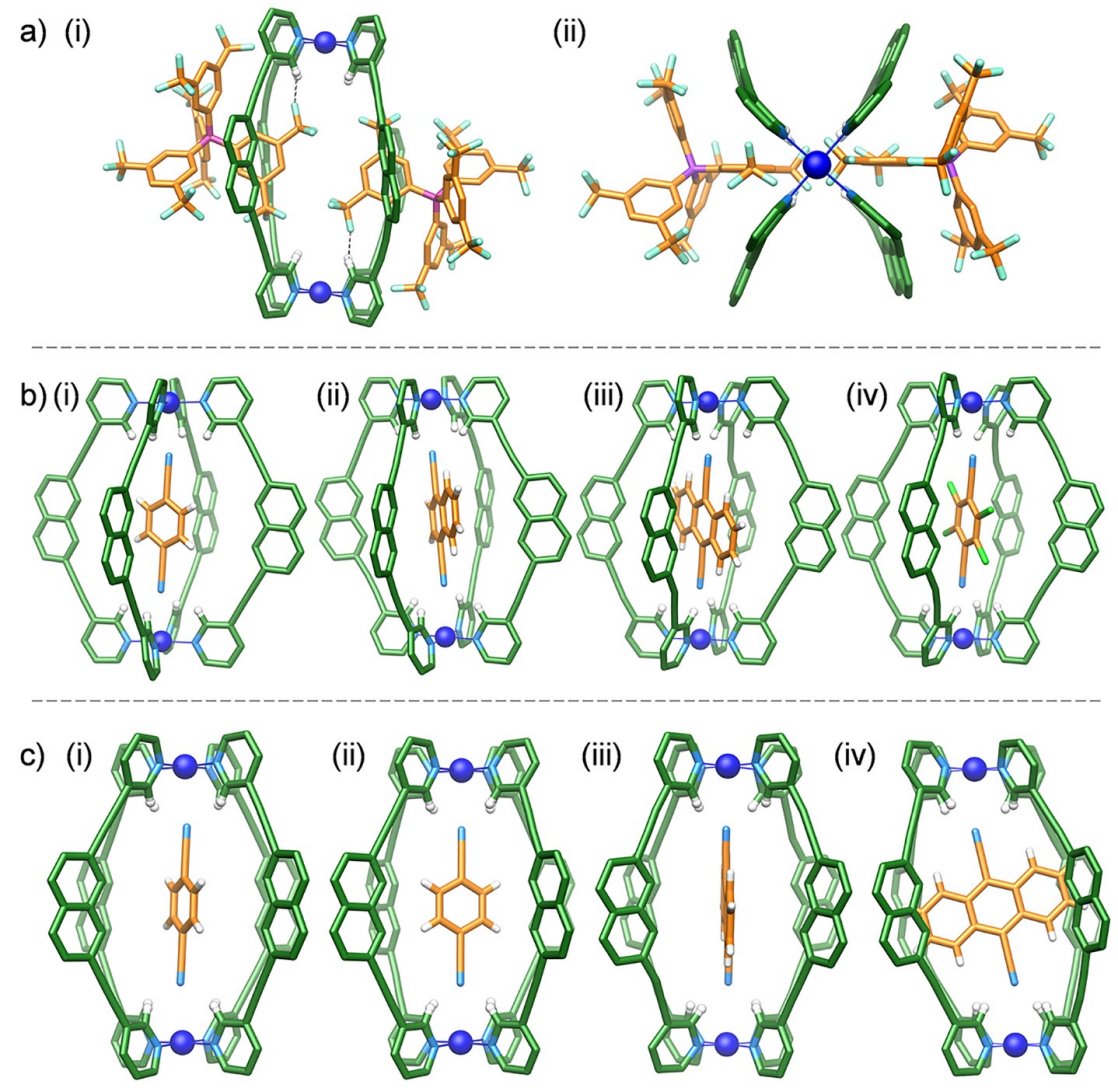
X-ray crystal structures of the cage structure **2** and dicyanoarene⊂**2** host–guest complexes. (a) “Empty” cage structure **2** showing the partial ingress of BArF counteranions, with close contacts represented by the dashed black lines. (b) Host–guest complexes: (i) **DCB**⊂**2**; (ii) **DCN**⊂**2**; (iii) **DCA**⊂**2**; (iv) **TCDCB**⊂**2**. (c) Alternative views of (i and ii) **DCB**⊂**2** and (iii and iv) **DCA**⊂**2**. The C atoms of the cage are shown in green, and the C atoms of the guests are shown in orange. Other color codes: Pd, blue; N, light blue; F, cyan; B, pink; H, white.

Cage **2** was characterized by a variety of NMR techniques (Figure S4) and electrospray ionization mass spectrometry (Figure S6). In addition, crystals suitable for single-crystal X-ray diffraction were grown from the diffusion of diethyl ether into a solution of **2** in CH_2_Cl_2_ over 2 days. The crystal structure of **2** (Figure 2a) is described by a Pd_2_L_4_ distorted lantern, whereby the two Pd^II^ ions are linked by the four 2,7-naphthalene-based ligands, providing a cavity with a Pd–Pd distance of ≈14 Å. As predicted, the interpalladium distance is slightly larger than that of cage **1** (≈12 Å).^27^ Interestingly, the structure also shows an unexpected feature: two BArF counterions are partially protruding into the cavity. These anions are interacting with the cage in the same manner; each forms interactions between the two CF_3_ groups from a single BArF aryl ring and the *o*-pyridyl H atoms that usually H-bond to carbonyl guests [Figure 2a(i)]. However, because each counteranion only partially occupies the cavity, they interact with just two H atoms at either site, with close contacts between ArF_2_C–F···H–Py of ≈2.5 Å. Globally, partial inclusion of the bulky anions distorts the overall structure, creating two larger portals at the expense of the other two and shifting the cage toward *D*_2*h*_ symmetry. This is most obvious when viewed down the Pd–Pd axis [Figure 2a(ii)]. Whether the same hydrogen-bonding interactions between the BArF anions and **2** are maintained in solution is difficult to gauge. However, it can be noted that the ^19^F NMR shift for cage **2** is very similar to that of other cages that we have previously made and also NaBArF, suggesting that in solution this interaction, at best, is very weak.

In order to probe the host–guest chemistry, we first utilized a more conventional approach using a combination of X-ray crystallography and ^1^H NMR spectroscopy. Four different commercially available guest compounds were investigated (Figure 1b): 1,4-dicyanobenzene (**DCB**), 1,4-dicyanonaphthalene (**DCN**), 9,10-dicyanoanthracene (**DCA**), and 2,3,5,6-tetrachlorodicyanobenzene (**TCDCB**). Single crystals were grown by either slow evaporation of an NMR sample or diffusion of diethyl ether into solutions of the host–guest complexes in CH_2_Cl_2_ over 2–4 days. In all cases, the dicyanoarene guest occupies the central cavity of the lantern, with the N atoms of the two cyano groups simultaneously interacting with the “top” and “bottom” pockets of the H-bond donors [Figure 2b(i)–(iv)]. The ArCN···H–Py H-bond distances cover a narrow range of 2.5–2.7 Å, while the ArCN···Pd distances span from 3.1 to 3.3 Å. For comparison, the equivalent C=O···H–Py H-bond and C=O···Pd distances in the pentacenedione⊂**1** structure that we have previously reported are 2.4–2.6 and 3.5 Å, respectively.^15^ The Pd–Pd distances of the dicyanoarene host–guest complexes are also close to that of **2**, ranging from 14.1 to 14.4 Å (**DCB**⊂**2** < **TCDCB**⊂**2** < **DCN**⊂**2** < **DCA**⊂**2**). There are, however, also some interesting features that appear to correlate with the solution host–guest chemistry (*vide infra*). The most obvious aspect relates to subtle distortions in the overall cage structure, which appears to relate to the way the guest is aligned within the cavity. In all of the structures, the two N_4_Pd coordination planes are aligned close to parallel. For **DCB**⊂**2**, the Pd ions connect the two planes vertically, with the **DCB** guest sitting along this vertical axis [Figure 2c(i),(ii)]. In contrast, the guest molecules in the structures of **DCN**⊂**2**, **DCA**⊂**2**, and **TCDCB**⊂**2** are aligned noticeably away from vertical, which is particularly evident when viewed through one set of opposing portals [Figure 2c(iii),(iv)]. This causes the overall structure to become distorted away from *D*_4*h*_ symmetry, with offset PdN_4_ coordination planes. Because this distortion is not apparent in **DCB**⊂**2** but is present in all of the structures with the larger guests, this would suggest that secondary interactions between the inward-facing naphthyl H atoms (H_g_) and the different guests may play a role in the preferred conformation that the cage adopts.

**Figure 3:**
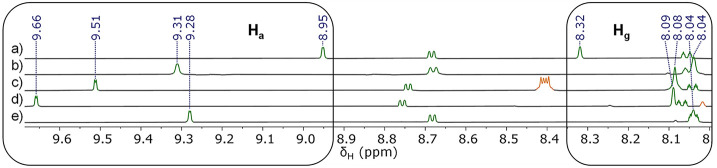
^1^H NMR (500 MHz, CD_2_Cl_2_, 300 K) spectra of cage **2** and dicyanoarene⊂**2** host–guest complexes. (a) ^1^H NMR spectrum of “empty” cage **2**. ^1^H NMR spectra of a mixture of cage **2** and (b) **DCB**, (c) **DCN**, (d) **DCA**, and (e) **TCDCB**. The cage and dicyanoarene guest signals are highlighted in green and orange, respectively. The lettering refers to the assignments shown in Figure 1.

A combination of ^1^H NMR and UV–vis experiments show that **DCB**, **DCN**, **DCA**, and **TCDCB** are also guests for **2** in solution. The ^1^H NMR spectra of the host–guest complexes are particularly informative; a pronounced downfield shift (0.3–0.7 ppm) of the inward-facing *o*-pyridyl proton of **2** (H_a_), which H-bonds to the N atom of the guests, is observed (Figure 3). Conversely, the inward-facing naphthyl protons (H_g_) become shielded (Figure 3), presumably because of interactions between these H atoms and the π surface of the guest. Association constants were determined by titration experiments using either ^1^H NMR (**DCB**⊂**2** and **DCN**⊂**2**) or UV–vis (**DCA**⊂**2**) spectroscopy. Unfortunately, no association constant for **TCDCB**⊂**2** could be obtained because of the onset of crystallization upon the addition of excess **TCDCB** to **2**. All of the measured nitrile guests show significant binding with **2** (≥10^3^ M^–1^).

A comparison of the association constants for dicyanoarene⊂**2** and quinone⊂**1** reveals some interesting trends and differences (Table S1). First, the dicyanoarene⊂**2** complexes are all weaker than quinone⊂**1** (e.g., benzoquinone⊂**1** vs **DCB**⊂**2**, etc.). This can possibly be explained by the poorer H-bond-accepting capacity of nitriles compared to ketones, as predicted by Hunter.^28^ The affinity of **2** toward dinitriles with extended π systems increases, i.e., **DCB** < **DCN** < **DCA**, which is also observed with quinone⊂**1**, where guest binding increases along the series benzoquinone, naphthoquinone, and anthraquinone. However, this effect is much less pronounced for dicyanoarene⊂**2**. For example, the *K*_a_ for **DCA**⊂**2** is just over one order of magnitude higher than that for **DCB**⊂**2**. In contrast, the difference in the affinity of benzoquinone and anthraquinone for **1** is approximately 10^4^. It has been suggested that the increased binding of these larger quinones in the case of **1** is due to the favorable secondary interactions between the four inward-facing “equatorial” CH groups of the cage and the extended π surface of the guest. It seems somewhat counterintuitive then that **2** possesses twice the number of “equatorial” CH groups that could interact with the guest yet shows weaker relative binding. This could possibly be caused by the need to optimally align these interactions, which could require the cage to distort, as has been observed with the single-crystal X-ray structures, causing the relative energy of the cage structure to increase and leading to a smaller increase in the affinity. Finally, we have also measured the affinity of **2** toward benzoquinone to demonstrate that the host–guest chemistry of **2** (and, by inference, **1**) correlates strongly with the ability of the guest’s H-bond-accepting atoms to simultaneously interact with both sets of *o*-pyridyl H-bond-donor pockets. The *K*_a_ value of just 18 M^–1^ for benzoquinone⊂**2** fully supports this hypothesis (Figure S15).

**Figure 4:**
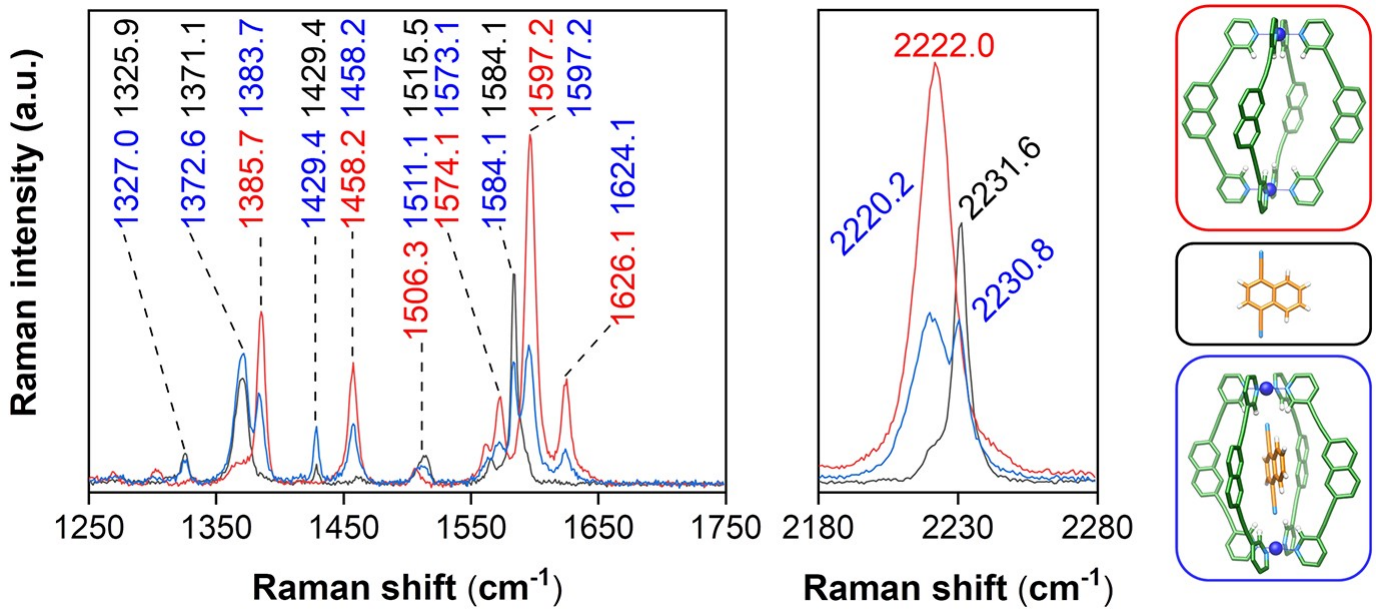
Solid-state analysis of the encapsulation of **DCN** within **2** using RS. Raman spectra were acquired from the free guest (**DCN**, black), “empty” cage (**2**, red), and host–guest complex (**DCN**⊂**2**, blue). Raman spectra were acquired using 785 nm excitation for 10 s with a 50× objective lens (0.18 mW). All assignments are in reciprocal centimeters. The full Raman spectra are provided in Figure S22.

RS was subsequently performed on milligram amounts of dried crystalline materials of all four host–guest complexes, the four free guests, **2**, and the cage ligand L. In order to ensure that the cage had not broken into its component parts, we first made comparisons between **2** and the free ligand L. For **2**, the ν(CC) (ring) stretching modes at 1626.1, 1574.1, 1458.2, and 1385.7 cm^–1^ were tentatively assigned to the naphthalene group (Figure 4),^29^ which all presented minor shifts in the Raman stretching frequencies compared to L (Figure S21). Evidence of coordination of the pyridyl moieties is confirmed by the large increase in the intensity and red-shifting of the Raman band assigned to the pyridine ring mode of L (1603.0 cm^–1^) compared to **2** (1597.2 cm^–1^). This is further corroborated by convergence of the two peaks assigned to the alkynyl stretching frequency in L (2206.9 and 2216.6 cm^–1^) into a single broad peak at 2222.0 cm^–1^ in **2**.

We next investigated the use of RS to probe the host–guest chemistry of **2** in both the solution and solid state. We will initially focus our discussion on the encapsulation of **DCN** as an exemplar because this was the second-highest-affinity guest and did not have any issues with regard to fluorescence preventing the acquisition of solution data, as was the case for **DCA**.

Analysis of the solid-state RS of **DCN**, **2**, and **DCN**⊂**2** showed spectral features indicative of encapsulation (Figure 4), particularly in the regions of 1250–1750 and 2180–2280 cm^–1^. First, the Raman spectrum of **DCN**⊂**2** shows a combination of peaks from both the host (e.g., 1458.2 and 1625.0 cm^–1^) and guest (e.g., 1327.0, 1429.4, and 1584.1 cm^–1^). Notably, however, many of the signals show stretching frequency shifts compared to “empty” cage **2** and free **DCN**. For example, the nitrile of **DCN** shifts from 2231.6 cm^–1^ in the free guest to 2230.8 cm^–1^ in the host–guest complex, with peaks in the fingerprint region also producing frequency shifts upon encapsulation (e.g., from 1325.9 to 1327.0 cm^–1^ and from 1515.5 to 1511.1 cm^–1^). The encapsulation of **DCN** also results in spectral shifts of the host, including a red-shifting of the alkynyl stretching frequency of the ligand (from 2222.0 to 2220.2 cm^–1^; Figure 4), with a corresponding reduction in the Raman scattering intensity. Of the naphthalene modes, those at 1626.1, 1574.1, and 1385.7 cm^–1^ shift upon encapsulation of **DCN**, while the band at 1458.2 cm^–1^ is generally unaffected by guest occupation, indicating the varying extents to which encapsulation distorts the lantern structure.

We have also made a general comparison of the spectral frequency differences between **2** and each of the host–guest complexes in the solid state (Table S2). In general, the four free guests show intense peaks arising from the CN stretching in the region 2230–2260 cm^–1^ in the Raman spectra (Figure S24). Interestingly, the Raman spectrum of **TCDCB** shows two partially resolved CN stretching peaks at 2222.9 and 2213.2 cm^–1^, which may be ascribed to the A and B stretching modes. [A previous spectroscopic study of **DCB** identified a predominant Raman band at 2218 cm^–1^ [A mode, ν(CN)], while in contrast, another stretching mode B ν(CN) was assigned to the IR band at 2220 cm^–1^. This mode has a small polarization value, which will result in weak (if any) Raman scattering. In **TCDCB**, we propose that these two modes are detected.^31^] Because of the highly fluorescent nature of **DCA**, we were unable to collect a full Raman spectrum; however, a partial Raman spectrum was acquired at 785 nm laser excitation that identified a CN stretching mode at 2220.8 cm^–1^. For the host–guest complexes, the naphthalene Raman bands at 1385 and 1626 cm^–1^ were consistently red-shifted compared to **2**, while the 1458 cm^–1^ Raman band was generally unaffected by guest encapsulation. In addition, the modes at 1597 and 1574 cm^–1^ were both red-shifted upon encapsulation of **DCN** and **TCDCB** but were both blue-shifted upon encapsulation of **DCA**. These results indicate that the Raman spectral shifting of the lantern structure is directly impacted by the size and chemical nature of the encapsulated guest molecule.

Last, we examined solution-state experiments, focusing on the alkyne–nitrile region of the RS, because these two groups cannot be directly detected using ^1^H NMR spectroscopy. Several spectra were recorded with increasing equivalents of **DCN** with respect to **2** (Figure S23). These experiments show that the alkyne stretching frequency of **2** shifts from 2221.0 to 2219.2 cm^–1^ upon the addition of 3.5 equiv of **DCN**. Significantly, no further shift is observed, even after the addition of 16 equiv of **DCN**, with only an increase in the intensity of the signal for free **DCN** (2231.5 cm^–1^). These results indicate that we are observing the binding and saturation of **2** at low equivalents of **DCN**, which is consistent with our NMR data. This represents the first example (to the best of our knowledge) of the detection of a host–guest coordination cage complex using RS.

## Conclusion

We have described the host–guest chemistry of a novel Pd_2_L_4_ cage compound. This metallosupramolecular structure shows selective and high-affinity binding toward a range of dicyanoarene compounds. A combination of X-ray crystallography and ^1^H NMR spectroscopy has provided insight into guest encapsulation, revealing subtle features that affect the relative affinities of different species. Further analysis using RS has shown that this can be a complementary and informative technique, exploiting reporter functional groups that are present in many different coordination assemblies. We continue to use these methods to probe the noncovalent chemistry of cage compounds, paving the way to new opportunities in various applications, from magnetism^30^ to catalysis.

## Supporting Information Available

All synthetic procedures, titration experiments, and X-ray crystallographic and RS data (PDF)
