# Supplementary material for: Utilizing Raman Spectroscopy as a Tool for Solid- and Solution-Phase Analysis of Metalloorganic Cage Host–Guest Complexes
Source: Inorg Chem. 2022 May 5;62(5):1827–32. doi: 10.1021/acs.inorgchem.2c00873 (PMC9906719; doi:10.1021/acs.inorgchem.2c00873)
Supplement: Supplementary file 1 — ic2c00873_si_001.pdf [file ic2c00873_si_001.pdf]

Supporting Information

Utilizing Raman Spectroscopy as a Tool for Solid and Solution Phase Analysis of Metallo-Organic Cage Host-Guest Complexes

Helen M. O'Connor,†⊥ William J. Tipping,§ Julia Vallejo,† Gary S. Nichol,† Karen Faulds,§ Duncan Graham,§ Euan K. Brechin*,† and Paul J. Lusby*,†*

†EaStCHEM School of Chemistry, The University of Edinburgh, David Brewster Road, Edinburgh, EH9 3FJ, UK.

§Pure and Applied Chemistry, Technology and Innovation Centre, University of Strathclyde, 99 George Street, Glasgow, G1 1RD, UK.

⊥Present Address: School of Chemistry and Trinity Biomedical Sciences Institute (TBSI), Trinity College Dublin, The University of Dublin, Dublin 2, Ireland.

Corresponding Authors

Paul J. Lusby; orcid.org/0000-0001-8418-5687; Email: Paul.Lusby@ed.ac.uk

Euan K. Brechin; orcid.org/0000-0002-9365-370X; Email: E.Brechin@ed.ac.uk

Duncan Graham; orcid.org/0000-0002-6079-2105; Email: duncan.graham@strath.ac.uk

Table of Contents

S1. Materials and Methods S3
S2. Synthesis and NMR Spectroscopic Characterization S3
S2.1.1 Synthesis of 2,7-bis(pyridin-3-ylethynyl)naphthalene (L) S3
S2.1.2 Synthesis of $[Pd_2L_4](OTf)_4$ S4
S2.1.3 Synthesis of $[Pd_2L_4](BArF)_4$ (**2**) S5
S2.1.4 NMR spectroscopic data for dicyanoarene guests S6
S2.2.1 Spectroscopic data for 2,7-bis(pyridin-3-ylethynyl)naphthalene (L) S7
S2.2.2 Spectroscopic data for $[Pd_2L_4](OTf)_4$ S8
S2.2.3 Spectroscopic data $[(CD_3)_2SO]$ for $[Pd_2L_4](BArF)_4$ (**2**) S9
S2.2.4 Spectroscopic data (CD_2Cl_2) for $[Pd_2L_4](BArF)_4$ (**2**) S10
S2.2.5 Spectroscopic data (CD_2Cl_2) for dicyanoarene guests S11
S3. Mass Spectrometry S12
S4. Host-Guest Studies S13
S4.1 Spectroscopic data for host-guest complexes S13
S4.2 Experimental details for association constant determination S13
S4.3 Individual NMR titration data S14
S4.3.1 **DCB** with **2** S14
S4.3.2 **DCN** with **2** S15
S4.3.3 **DCA** with **2** S16
S4.3.4 Benzoquinone with **2** S17
S5. X-ray Crystallography S18
S5.1 General experimental details S18
S5.2 Crystallographic data and special refine details S18
S5.2.1 L S18
S5.2.2 **2** S18
S5.2.3 **DCB⊂2** S19
S5.2.4 **DCN⊂2** S19
S5.2.5 **DCA⊂2** S20
S5.2.6 **TCDCB⊂2** S20
S5.3 Crystallographic packing S21
S6. Raman Spectroscopy S23
S7. References S28

S1. Materials and Methods

All reagents and solvents were purchased from Alfa Aesar, VWR, Fluorochem, or Sigma Aldrich and used without further purification unless stated otherwise. All reactions were carried out under air, unless stated otherwise. All ^{1}H, ^{13}C and ^{19}F NMR spectra were recorded on either a 500 MHz Bruker AV III equipped with a DCH cryo-probe (Ava500), a 500 MHz Bruker AV IIIHD equipped with a Prodigy cryo-probe (Pro500), or a 400 MHz Bruker AV III equipped with BBFO+ probe (Ava400) at a constant temperature of 300 K. All DOSY experiments were performed on the Ava500 using bipolar gradient pulses for diffusion with two spoil gradients (ledbpg2s.compensated) pulse sequence. The sequence was carried out under automated conditions where the duration of the magnetic field pulse gradient (δ) was 1.5 ms and the diffusion time (Δ) was 100 ms. Typically, in each PFG NMR experiment, a series of 16 spectra on 32 K data points were collected and the eddy current delay (Te) was set to 5 ms in all experiments. The pulse gradients (g) were incremented from 2 to 95% of the maximum gradient strength in a linear ramp. The temperature was set and controlled at 300 K with an air flow of 400 L h^{-1} in order to avoid any temperature fluctuations due to sample heating during the magnetic field pulse gradients. Chemical shifts are reported in parts per million. For ^{1}H NMR spectra chemical shifts are referenced to 2.50 and 5.32 ppm for dimethyl sulfoxide-d_6 [$(CD_3)_2SO$] and methylene chloride-d_2 (CD_2Cl_2), respectively. For ^{13}C NMR spectra chemical shifts are referenced to 39.52 and 53.84 ppm for dimethyl sulfoxide-d_6 and methylene chloride-d_2, respectively. Apparent multiplicities are reported using the following standard abbreviations: m = multiplet, q = quartet, t = triplet, d = doublet, s = singlet, bs = broad singlet. All NMR spectroscopic analysis was performed with MestReNova, Version 14.

S2. Synthesis and NMR Spectroscopic Characterization

S2.1.1 Synthesis of 2,7-bis(pyridin-3-ylethynyl)naphthalene (L)

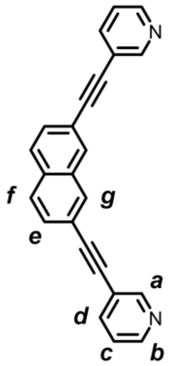

L was synthesized according to a modified literature procedure.[S1]

To a solution of 2,7-dibromonaphthalene (2.86 g, 10 mmol) and 3-ethynylpridine (2.50 g, 24 mmol) in degassed NEt_3 (150 mL) was added [$Pd(PPh_3)_2Cl_2$] (0.56 g, 8 mol%) and [CuI] (0.24 g, 15 mol%). The solution was stirred for 3 days at 80 °C under an inert atmosphere during which time a brown precipitate formed. The solvent was removed *in vacuo* before CH_2Cl_2 (150 mL) was added and the solution filtered. The filtrate was washed with saturated aqueous sodium bicarbonate (40mL) and H_2O (2 x 40 mL) before the organic layer was collected, dried over anhydrous $MgSO_4$, and the solvent removed *in vacuo*. The residue was purified by column chromatography on silica gel (CH_2Cl_2:acetone gradient 100:0 to 50:50) to yield L as a crystalline yellow-brown product. Yield = 2.16 g, 6.6 mmol, 66%.

Single X-ray quality crystals were grown from a standing solution of L in $(CD_3)_2SO$. Crystallographic details are provided in section S5.2.

^{1}H NMR (500 MHz, $(CD_3)_2SO$): δ 8.84 (2 H, bs, Ha), 8.64 (2 H, bs, Hb), 8.28 (2 H, s, Hg), 8.04 (4 H, m, Hd and Hf), 7.72 (2 H, dd, J = 8.5, 1.6 Hz, He), 7.51 (2 H, m, Hc) ppm.

^{1}H NMR (500 MHz, (CD_2Cl_2): δ 8.81 (2 H, s, Ha), 8.57 (2 H, d, J = 4.4 Hz, Hb), 8.09 (2 H, m, Hg), 7.87 (2 H, dt, J = 7.9, 1.9 Hz, Hd), 7.86 (2 H, m, Hf), 7.64 (2 H, dd, J = 8.6, 1.5 Hz, He), 7.32 (2 H, ddd, J = 7.9, 4.9, 0.7 Hz, Hc) ppm.

^{13}C NMR (126 MHz, $(CD_3)_2SO$): δ 151.7, 149.2, 138.6, 132.21, 132.18, 131.4, 129.2, 128.5, 123.8, 120.1, 119.3, 92.2, 87.1 ppm.

^{1}H DOSY NMR (500 MHz, $(CD_3)_2SO$): 2.76 × 10^{-10} m^2/s, hydrodynamic radius = 3.4 Å.

S2.1.2 Synthesis of $[Pd_2L_4](OTf)_4$

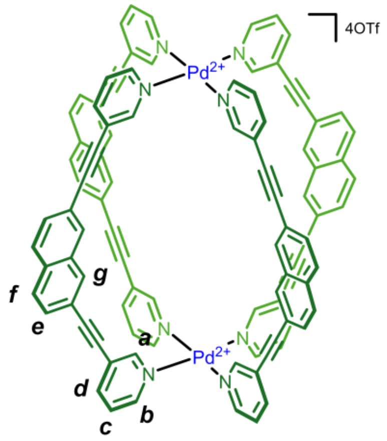

To a solution of L (1.038 g, 3.14 mmol) in CH_2Cl_2 (60 mL) was added a solution of $[Pd(CH_3CN)_4](OTf)_2$ (0.636 g, 1.57 mmol) in CH_3CN (60 mL) dropwise. The solution was stirred for 1 hour after which time the solvent was reduced *in vacuo* to approximately 5 mL. Diethyl ether (100 mL) was used to precipitate $[Pd_2L_4](OTf)_4$ as an off-white solid which was collected, washed with diethyl ether (20 mL), and dried under reduced pressure. Yield = 1.290 g, 0.61 mmol, 78%.

^{1}H NMR (500 MHz, $(CD_3)_2SO$): δ 9.63 (8 H, d, J = 1.7 Hz, Ha), 9.41 (8 H, dd, J = 5.8, 0.8 Hz, Hb), 8.42 (8 H, s, Hg), 8.31 (8 H, dt, J = 8.0, 1.4 Hz, Hd), 8.04 (8 H, d, J = 8.7 Hz, Hf), 7.85 (8 H, dd, J = 7.9, 6.0 Hz, Hc), 7.72 (8 H, dd, J = 8.3, 1.4 Hz, He) ppm.

^{13}C NMR (126 MHz, $(CD_3)_2SO$): δ 152.5, 150.4, 142.8, 132.9, 132.3, 131.8, 129.5, 128.9, 127.4, 122.4, 120.8 (q, $J_{C\text{-}F}$ = 322 Hz), 119.3, 94.9, 85.2 ppm.

^{19}F NMR (471 MHz, $(CD_3)_2SO$): δ −77.57 ppm.

^{1}H DOSY NMR (500 MHz, $(CD_3)_2SO$): 1.09 × 10^{-10} m^2/s, hydrodynamic radius = 10.0 Å.

S2.1.3 Synthesis of $[Pd_2L_4](BArF)_4$ (**2**)

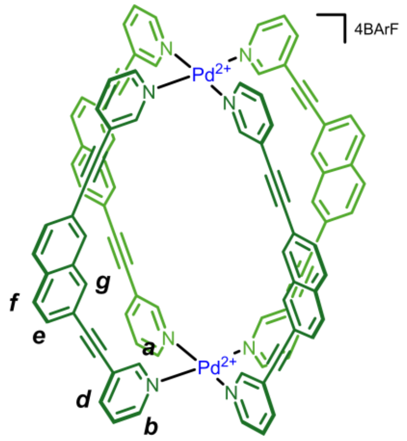

$[Pd_2L_4](OTf)_4$ (97 mg, 0.04 mmol) and NaBArF (164 mg, 0.19 mmol) were placed into CH_2Cl_2 (20 mL). The solution was sonicated for 30 minutes before it was filtered, and the solvent removed *in vacuo*. CH_3CN (2 mL) was used to dissolve the product before water (20 mL) was used to precipitate **2** as an off-white solid, which was collected, washed with water (5 mL), and dried under reduced pressure. Yield = 171 mg, 0.03 mmol, 75%.

Single X-ray quality crystals were grown from vapor diffusion of diethyl ether into a solution of **2** in CH_2Cl_2 over 2 days.

^{1}H NMR (500 MHz, $(CD_3)_2SO$): δ 9.62 (8 H, d, J = 1.6 Hz, Ha), 9.40 (8 H, dd, J = 5.8, 1.0 Hz, Hb), 8.48 (8 H, s, Hg), 8.29 (8 H, dt, J = 8.1, 1.4 Hz, Hd), 8.03 (8 H, d, J = 8.4 Hz, Hf), 7.85 (8 H, dd, J = 8.0, 6.0 Hz, Hc), 7.70 (24 H, m, HBArF and He), 7.61 (32 H, t, J = 2.3 Hz, HBArF) ppm.

^{1}H NMR (500 MHz, CD_2Cl_2): δ 8.95 (8 H, d, J = 1.6 Hz, Ha), 8.68 (8 H, dd, J = 6.0, 0.9 Hz, Hb), 8.32 (8 H, s, Hg), 8.06 (8 H, dt, J = 8.1, 1.5 Hz, Hd), 7.87 (8 H, d, J = 8.5 Hz, Hf), 7.70 (32 H, t, J = 2.5 Hz, HBArF), 7.63 (8 H, dd, J = 8.5, 1.5 Hz, He), 7.54 (8 H, dd, J = 8.1, 6.0 Hz, Hc), 7.51 (16 H, s, HBArF) ppm.

^{13}C NMR (126 MHz, $(CD_3)_2SO$): δ 161.1 (q, $J_{C\text{-}B}$ = 50 Hz), 152.5, 150.5, 142.8, 134.0, 132.9, 132.5, 131.8, 129.5, 128.6 (q, $J_{C\text{-}F}$ = 32 Hz), 127.4, 124.0 (q, $J_{C\text{-}F}$ = 272 Hz), 122.4, 119.2, 118.0, 117.7 (m), 94.9, 85.2 ppm.

^{13}C NMR (126 MHz, CD_2Cl_2): δ 162.1 (q, $J_{C\text{-}B}$ = 50 Hz), 151.7, 148.4, 144.8, 135.2, 134.5, 132.6, 132.4, 130.2, 129.3 (q, $J_{C\text{-}F}$ = 32 Hz), 126.8, 125.0 (q, $J_{C\text{-}F}$ = 272 Hz), 119.6, 118.3, 117.9 (m), 98.9, 83.4 ppm. Only 12 ligand carbon signals can be identified, which we attribute to either overlapping or the low intensity of the missing resonance.

^{19}F NMR (471 MHz, $(CD_3)_2SO$): δ −61.61 ppm.

^{19}F NMR (471 MHz, CD_2Cl_2): δ −62.72 ppm.

^{1}H DOSY NMR (500 MHz, $(CD_3)_2SO$): 1.04×10^{-10} m^2/s, hydrodynamic radius = 10.5 Å.

^{1}H DOSY NMR (500 MHz, CD_2Cl_2): 4.18×10^{-10} m^2/s, hydrodynamic radius = 12.6 Å.

S2.1.4 NMR spectroscopic data for dicyanoarene guests

All guests were purchased from Sigma Aldrich or Fluorochem and used without further purification. Multinuclear NMR spectroscopy was used to confirm the purity of the guests which were compared against literature values where possible.

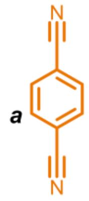

1,4-Dicyanobenzene (DCB): ^{1}H NMR (500 MHz, CD_2Cl_2): δ 7.80 (4 H, s, Ha) ppm.[S2]

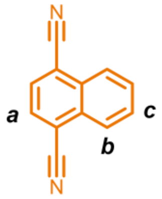

1,4-Dicyanonaphthalene (DCN): ^{1}H NMR (500 MHz, CD_2Cl_2): δ 8.35 (2 H, dd, *J* = 6.4, 3.3 Hz, Hb), 8.70 (2 H, s, Ha), 7.88 (2 H, dd, *J* = 6.4, 3.2 Hz, Hc) ppm.[S3]

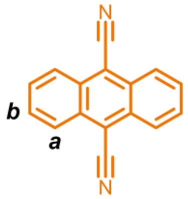

9,10-Dicyanoanthracene (DCA): ^{1}H NMR (500 MHz, CD_2Cl_2): δ 8.53 (4 H, dd, *J* = 6.7, 3.2 Hz, Ha), 7.87 (4 H, dd, *J* = 6.7, 3.2 Hz, Hb) ppm.[S4]

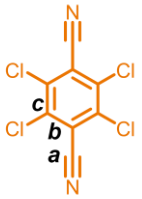

2,3,5,6-Tetrachlorodicyanobenzene (TCDCB): ^{13}C NMR (126 MHz, CD_2Cl_2): δ 136.7 (Cc), 120.5 (Cb), 112.2 (Ca) ppm.

S2.2.1 Spectroscopic data for 2,7-bis(pyridin-3-ylethynyl)naphthalene (L)

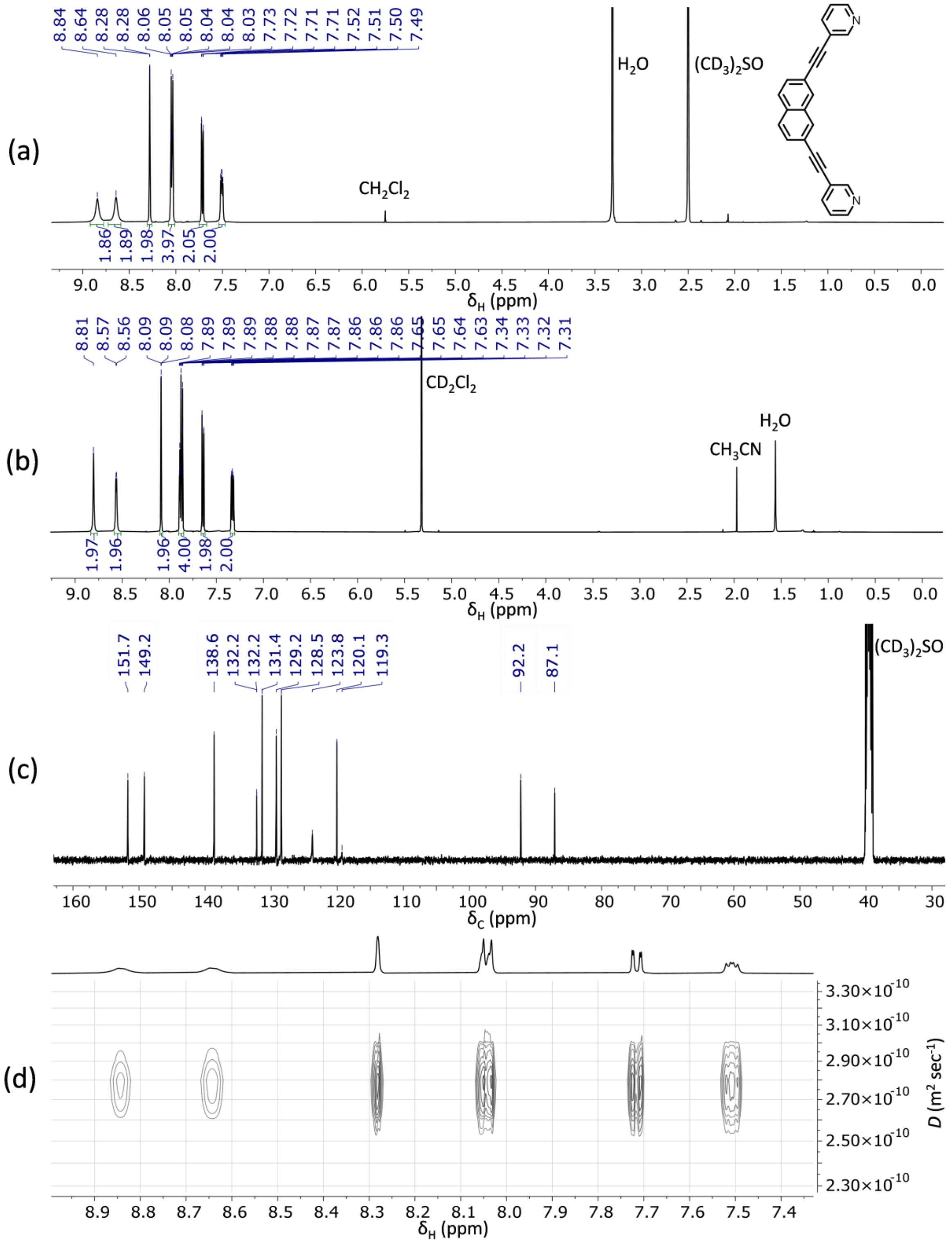

Figure S1 (a) ^{1}H NMR spectrum [$(CD_3)_2SO$, 500 MHz], (b) ^{1}H NMR spectrum (CD_2Cl_2, 500 MHz), (c) ^{13}C NMR spectrum [$(CD_3)_2SO$, 126 MHz], and (d) ^{1}H DOSY NMR spectrum [$(CD_3)_2SO$, 500 MHz] of L.

S2.2.2 Spectroscopic data for $[Pd_2L_4](OTf)_4$

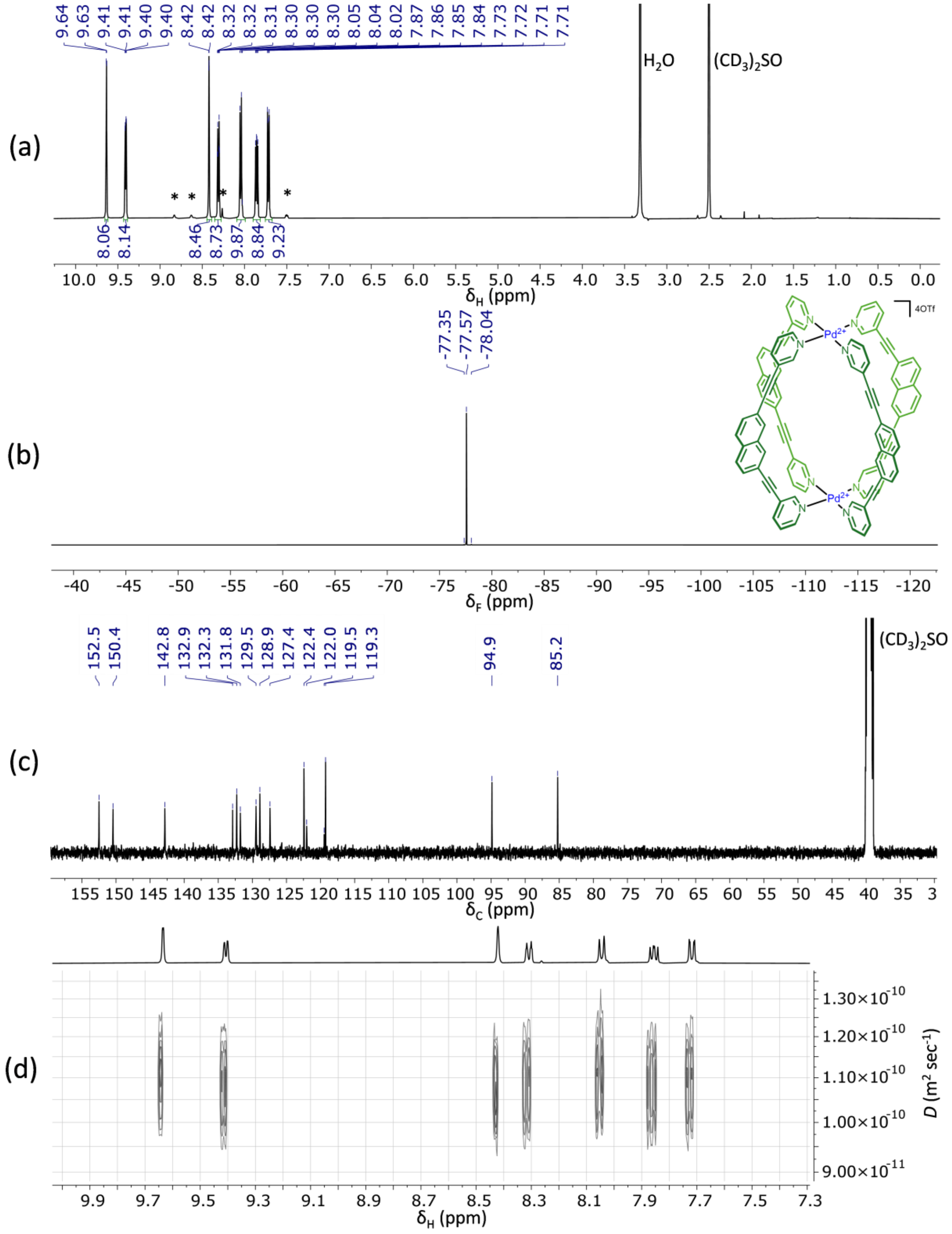

Figure S2 (a) ^{1}H NMR spectrum [$(CD_3)_2SO$, 500 MHz], (b) ^{19}F NMR spectrum [$(CD_3)_2SO$, 471 MHz], (c) ^{13}C NMR spectrum [$(CD_3)_2SO$, 126 MHz], and (d) ^{1}H DOSY NMR spectrum [$(CD_3)_2SO$, 500 MHz] of $[Pd_2L_4](OTf)_4$. The star symbols in the ^{1}H NMR spectrum (*) denote excess ligand.

S2.2.3 Spectroscopic data [$(CD_3)_2SO$] for [Pd_2L_4]$(BArF)_4$ (**2**)

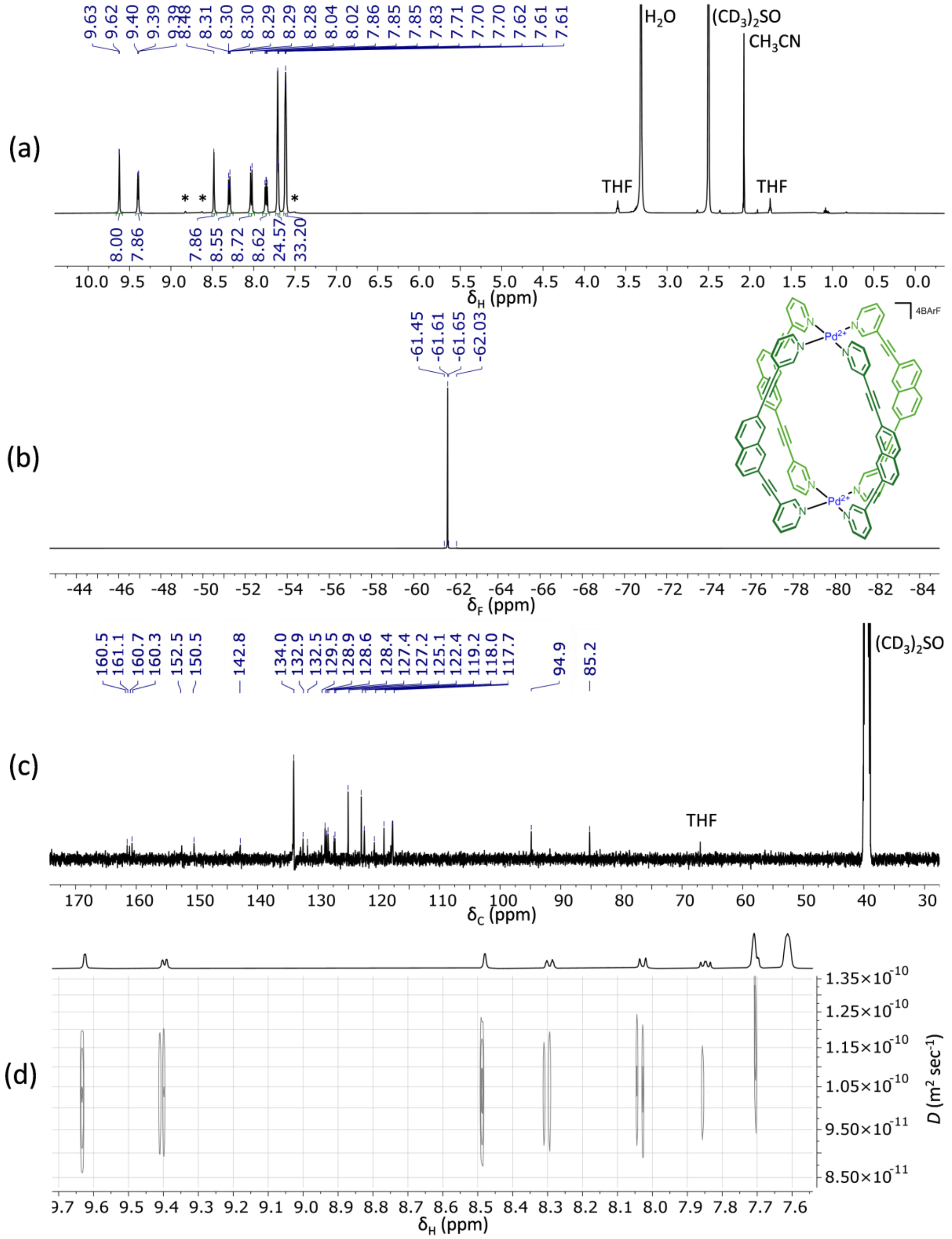

Figure S3 (a) ^{1}H NMR spectrum [$(CD_3)_2SO$, 500 MHz], (b) ^{19}F NMR spectrum [$(CD_3)_2SO$, 471 MHz], (c) ^{13}C NMR spectrum [$(CD_3)_2SO$, 126 MHz], and (d) ^{1}H DOSY NMR spectrum [$(CD_3)_2SO$, 500 MHz] of **2**. The star symbols in the ^{1}H NMR spectrum (*) denote excess ligand.

S2.2.4 Spectroscopic data (CD_2Cl_2) for $[Pd_2L_4](BArF)_4$ (**2**)

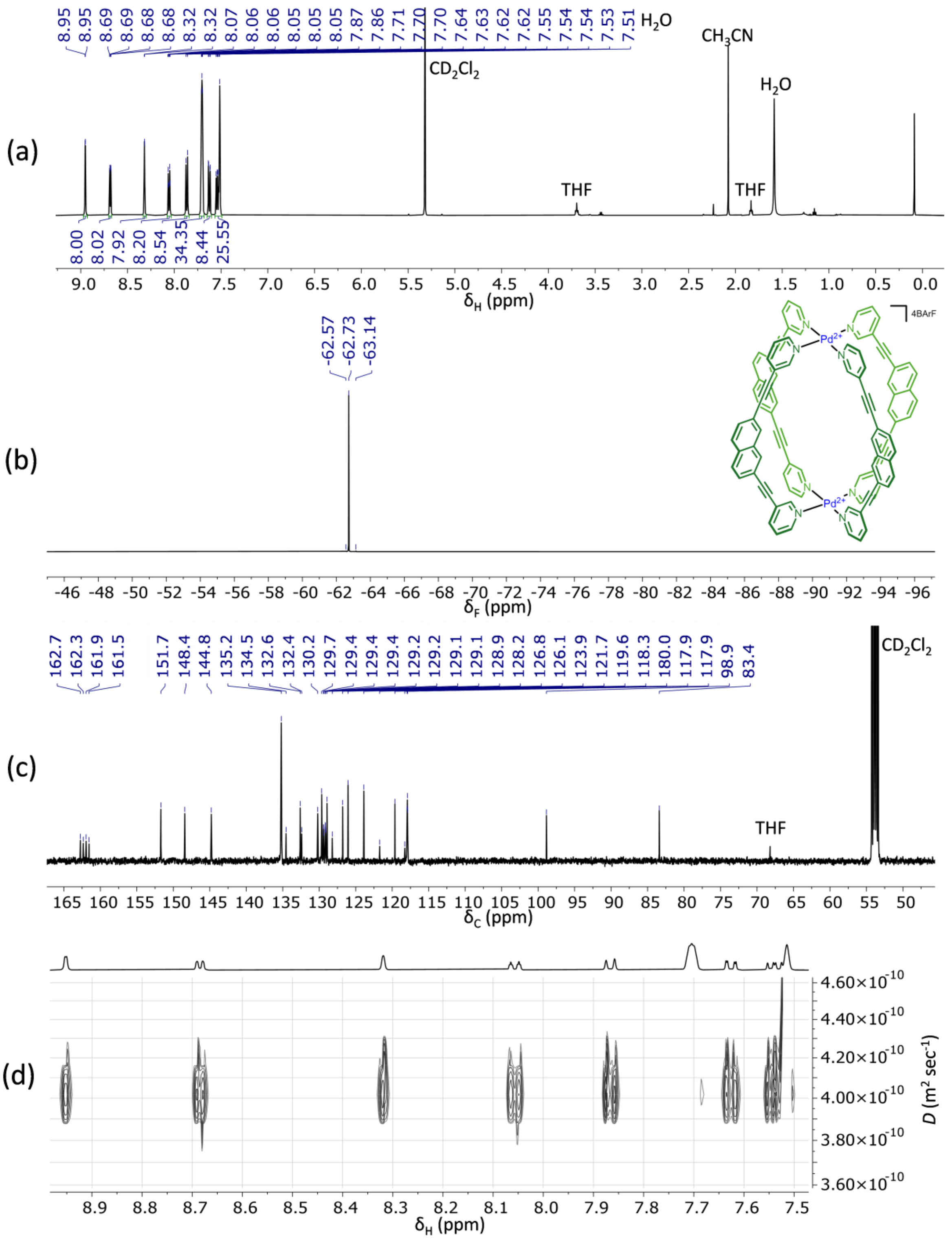

Figure S4 (a) ^{1}H NMR spectrum (CD_2Cl_2, 500 MHz), (b) ^{19}F NMR spectrum (CD_2Cl_2, 471 MHz), (c) ^{13}C NMR spectrum (CD_2Cl_2, 126 MHz), and (d) ^{1}H DOSY NMR spectrum (CD_2Cl_2, 500 MHz) of **2**.

S2.2.5 Spectroscopic data (CD_2Cl_2) for dicyanoarene guests

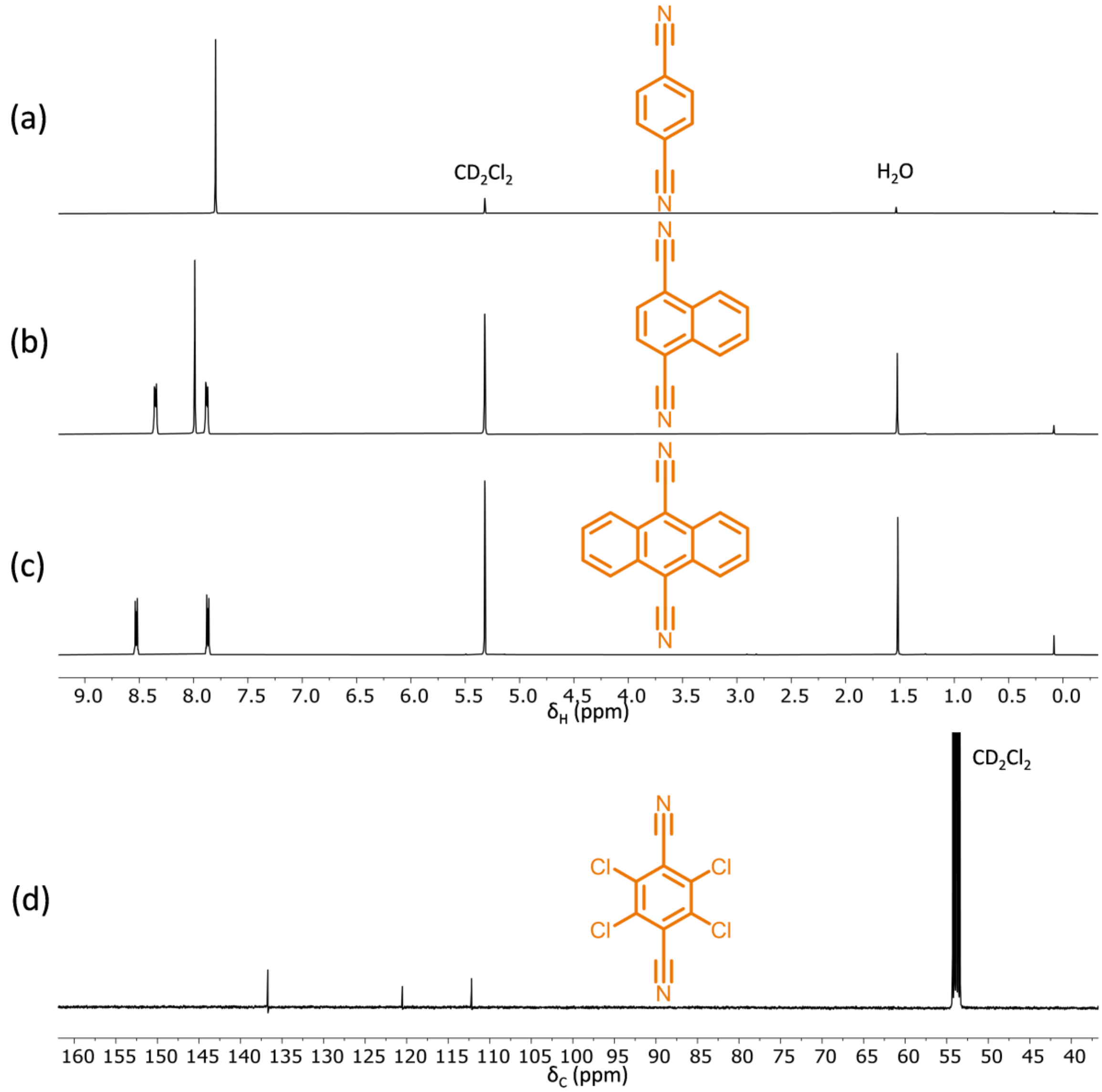

Figure S5 ^{1}H NMR spectra (CD_2Cl_2, 500 MHz) of (a) 1,4-dicyanobenzene (**DCB**), (b) 1,4-dicyanonaphthalene (**DCN**), and (c) 9,10-dicyanoanthracene (**DCA**). (d) ^{13}C NMR spectrum (CD_2Cl_2, 126 MHz) of 2,3,5,6-tetrachlorodicyanobenzene (**TCDCB**).

S3. Mass Spectrometry

Electrospray Ionization (ESI) mass spectra of **2** was performed on a Synapt G2 (Waters, Manchester, UK) mass spectrometer, using a direct infusion electrospray ionization source (ESI), controlled using MassLynx S5 v4.1 software. Crystals of **2** were collected and dried before the sample was dissolved in acetonitrile at 50 µM prior to the measurement. Capillary voltages were adjusted between 1.5 and 2.5 kV to optimize spray quality, while the sampling cone and the extraction cone voltage were minimized to reduce breakdown of the assemblies. Source temperature was set at 80 °C. The data was analyzed using the MassLynx v4.1 software, with predicted isotopic distributions calculated using mMass open-source mass spectrometry tool.[S5]

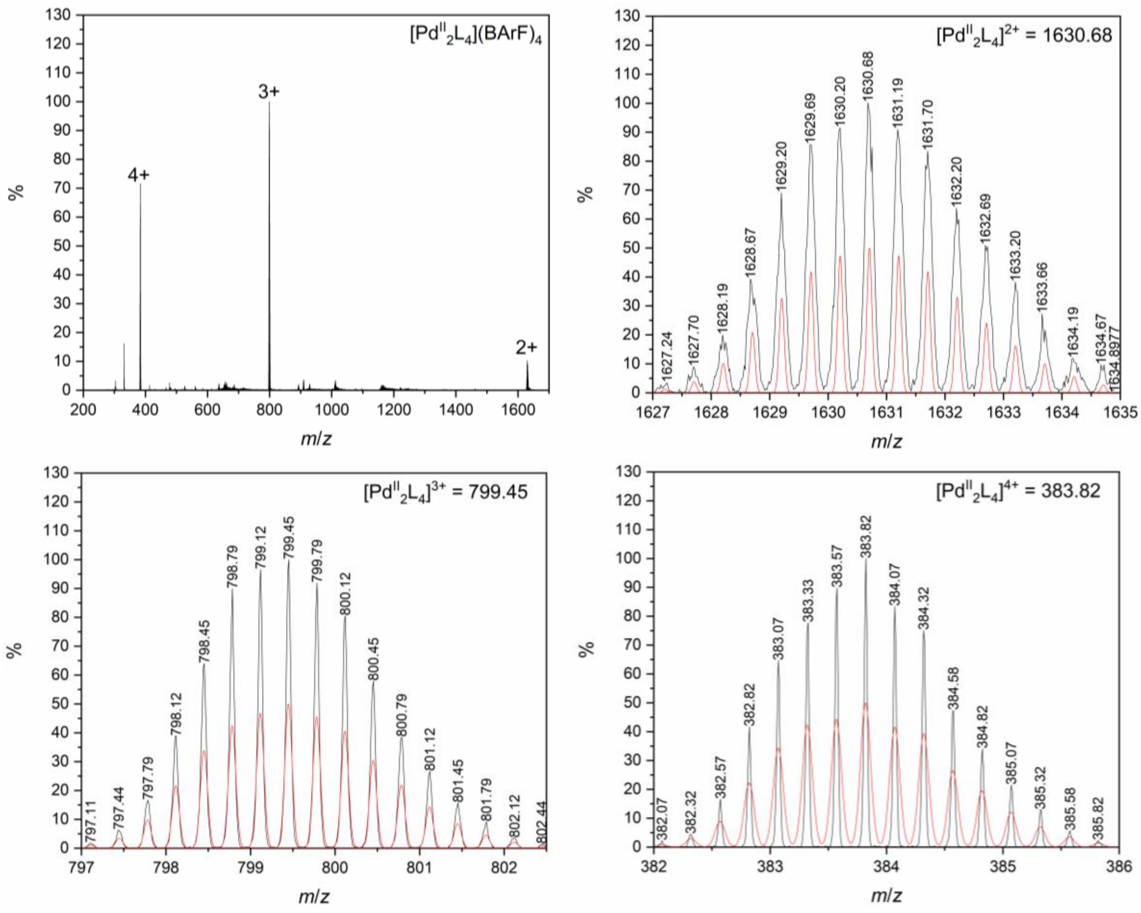

Figure S6 Mass spectrum of **2** with experimental results and modelled isotopic distributions shown in black and red, respectively.

S4. Host-Guest Studies

S4.1 Spectroscopic data for host-guest complexes

^{1}H NMR host-guest studies were performed on a 500 MHz Bruker AV III equipped with a DCH cryo-probe (Ava500) at 300 K. Initial sample volumes were 500 µL with a 0.45–0.50 mM concentration of **2** in CD_2Cl_2. The guests were added in excess as solids and the tubes sonicated for 5 minutes before ^{1}H NMR spectra were recorded.

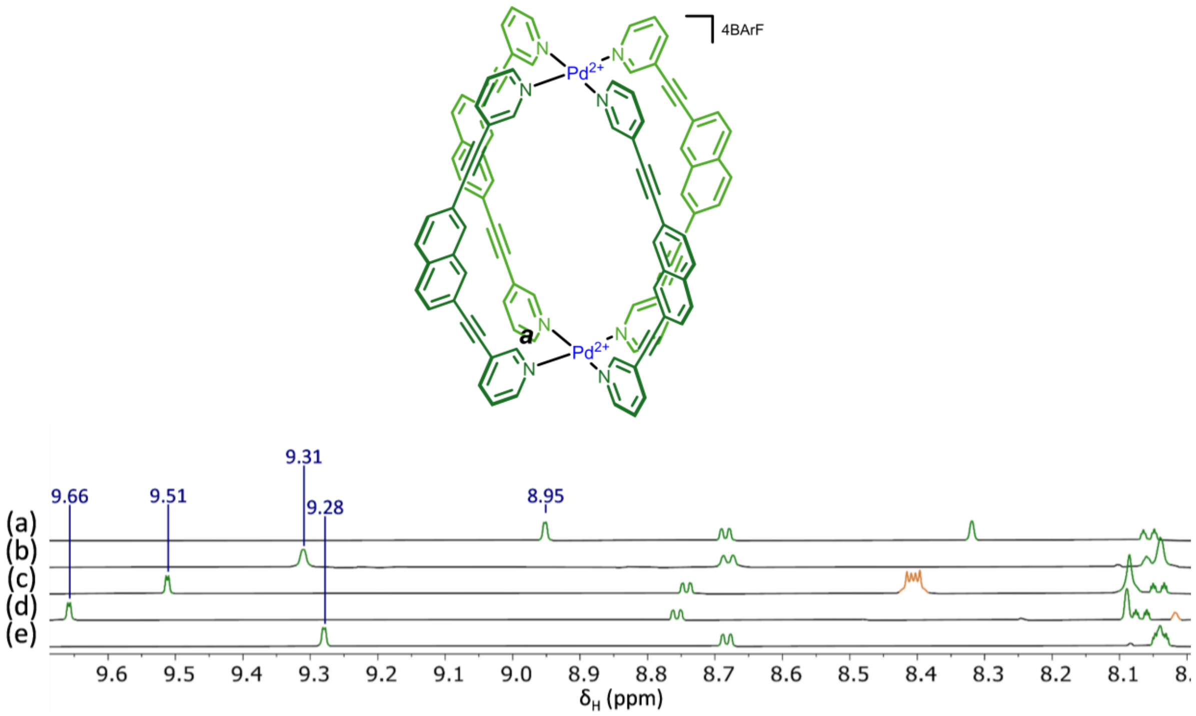

Figure S7 ^{1}H NMR host-guest spectra (500 MHz, CD_2Cl_2, 300 K) showing the shifting of Ha (labelled) of (a) **2** on the addition of (b) 1,4-dicyanobenzene (**DCB**), (c) 1,4-dicyanonaphthalene (**DCN**), (d) 9,10-dicyanoanthracene (**DCA**), (e) 2,3,5,6-tetrachlorodicyanobenzene (**TCDCB**). The host and guest signals are represented by the green and orange colors, respectively.

S4.2 Experimental details for association constant determination

^{1}H NMR titration experiments were carried out on a 400 MHz Bruker AV III spectrometer equipped with BBFO+ probe (Ava400) at 300 K. Initial sample volumes were 500 µL with 0.45–0.50 mM concentration of **2**. Solutions of the guest quinones were 15–30 mM in the same stock solution of the cage. ^{1}H NMR spectra were recorded at 0–30 equivalents of the dicyanoarene. Association constants were obtained by analysis of the resulting titration data using the 1:1 host–guest stoichiometry equation 1 for fast exchange using the Levenberg-Marquardt Nonlinear Least-Squares Algorithm implemented in the R software and the RStudio software interface.[S6] The error of the determined association constants are estimated to be less than 10%. UV-Vis spectroscopy was carried out on a JASCO V-670 Spectrophotometer running Spectra Manager II (Jasco). The data was analyzed and plotted using Origin 2018 software using equation 1. All measurements were made at room temperature (16–21 °C) at 100 µM in CH_2Cl_2 using a fused silica cuvette with a 10 mm path length.

$$\delta = \delta_0 + \frac{\Delta\delta_{Max}}{2}\left(\frac{c_{Guest}}{c_{Host}} + \frac{1}{c_{Host}\cdot K_{Ass}} + 1 - \sqrt{\left(\frac{c_{Guest}}{c_{Host}} + \frac{1}{c_{Host}\cdot K_{Ass}} + 1\right)^2 - \frac{4\cdot c_{Guest}}{c_{Host}}}\right) \quad \text{(Equation 1)}$$

S4.3 Individual NMR titration data

S4.3.1 **DCB** with **2**

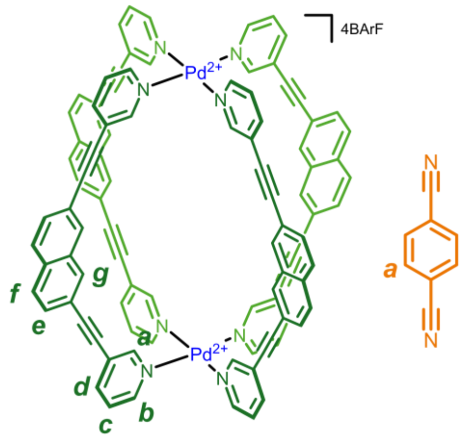

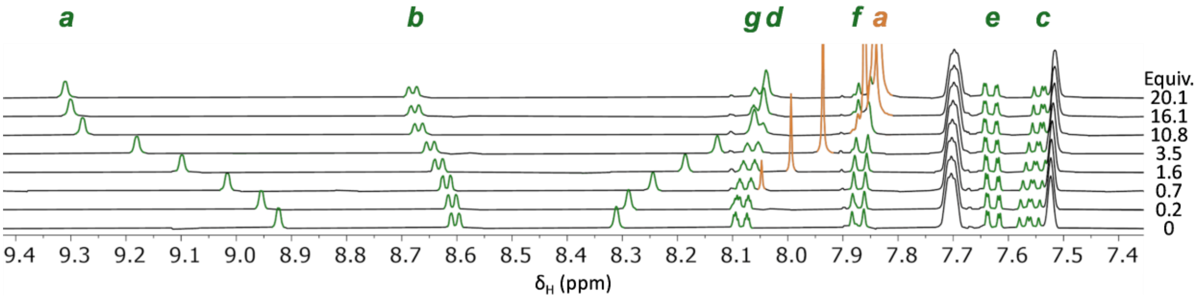

Figure S8 Partial ^{1}H NMR spectra (400 MHz, CD_2Cl_2, 300 K) of the titration of **2** (0.50 mM) with **DCB** (25 mM).

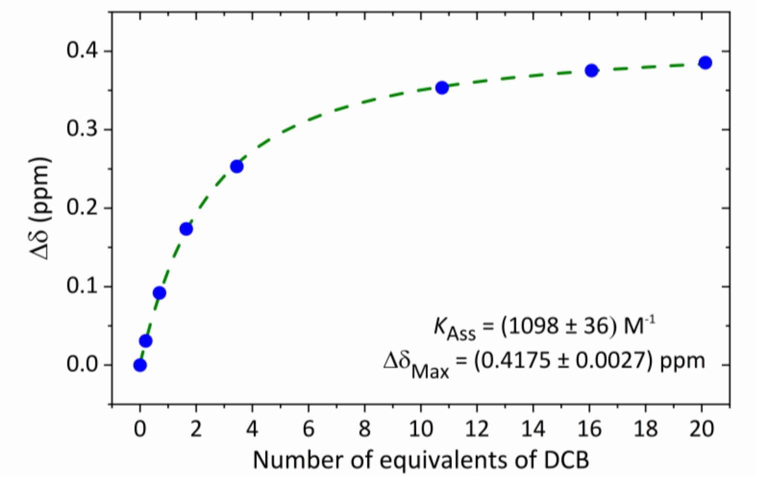

Figure S9 ^{1}H NMR (400 MHz, CD_2Cl_2, 300 K) titration curve of **2** (0.5 mM) with **DCB** (25 mM). The curve was obtained by monitoring the internal cage cavity proton Ha. The solid points represent the experimental data with the continuous dashed line represents the best-fit binding isotherm.

S4.3.2 **DCN** with **2**

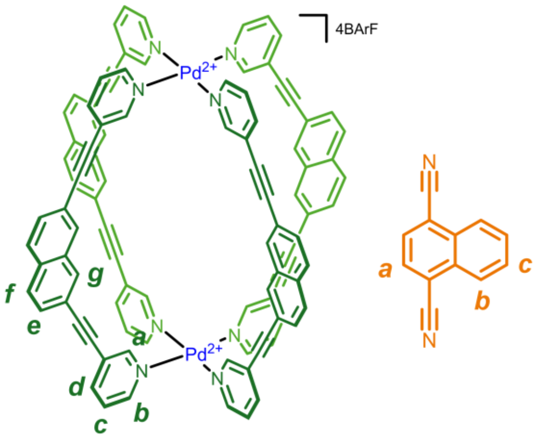

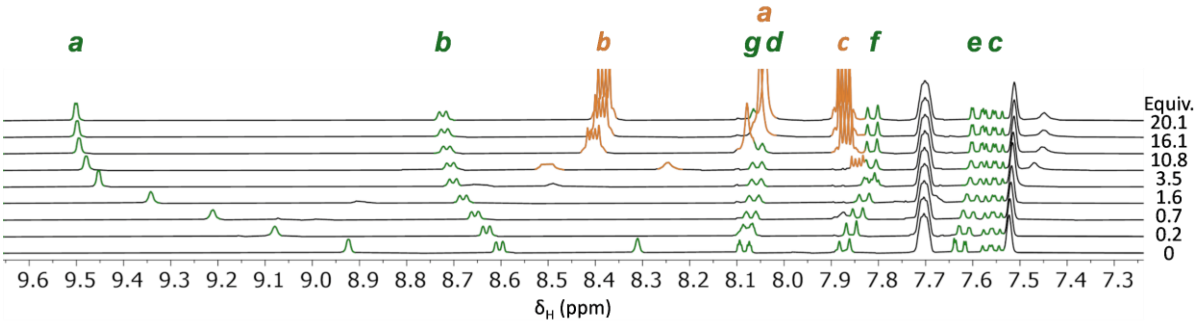

Figure S10 Partial ^{1}H NMR spectra (400 MHz, CD_2Cl_2, 300 K) of the titration of **2** (0.50 mM) with **DCN** (25 mM).

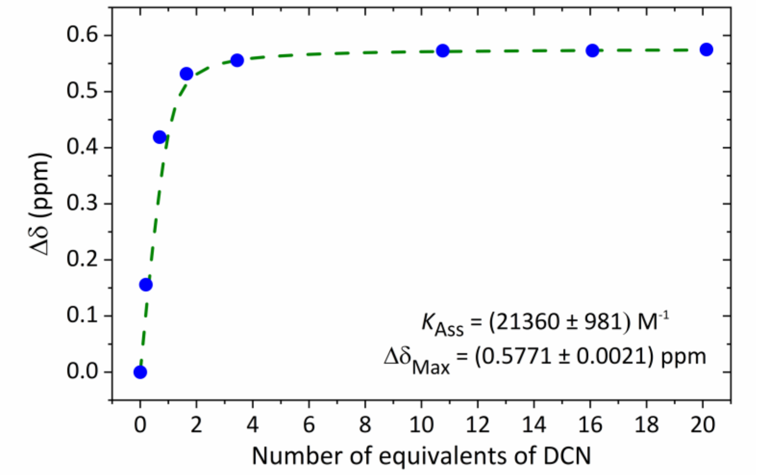

Figure S11 ^{1}H NMR (400 MHz, CD_2Cl_2, 300 K) titration curve of **2** (0.5 mM) with **DCN** (25 mM). The curve was obtained by monitoring the internal cage cavity proton Ha. The solid points represent the experimental data with the continuous dashed line represents the best-fit binding isotherm.

S4.3.3 **DCA** with **2**

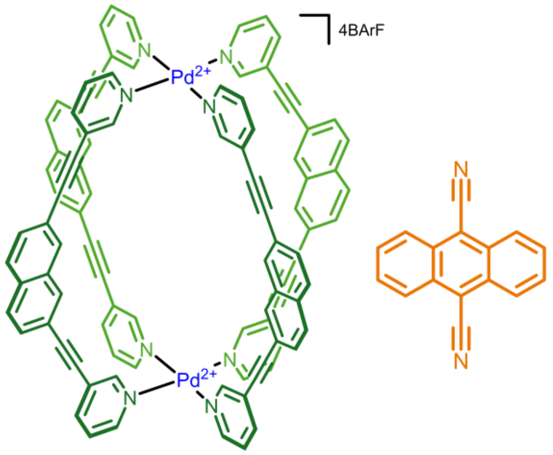

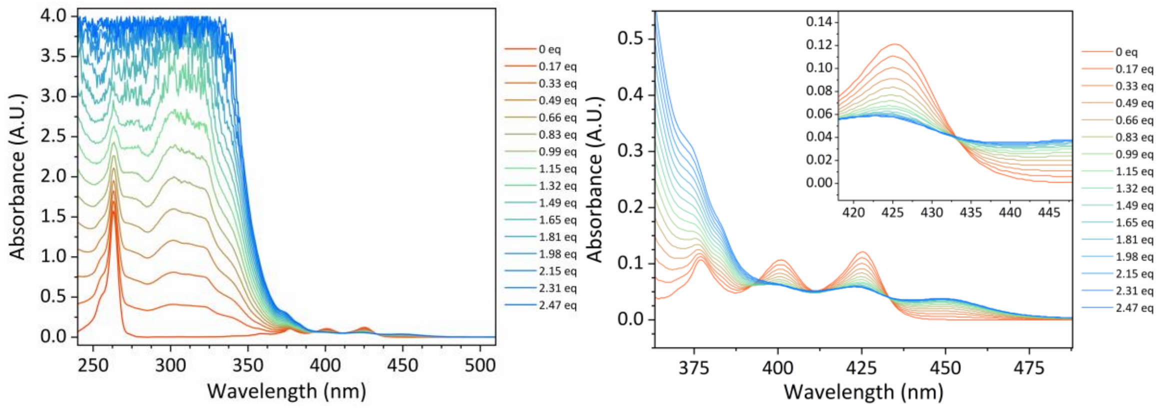

Figure S12 Full (left) and partial (right) UV-Vis spectrum of the titration of **2** (0.5 mM) with **DCA** (0.2 mM) in CH_2Cl_2, with the isosbestic point (right, inset) used for the determination of the binding constant (Figure S13).

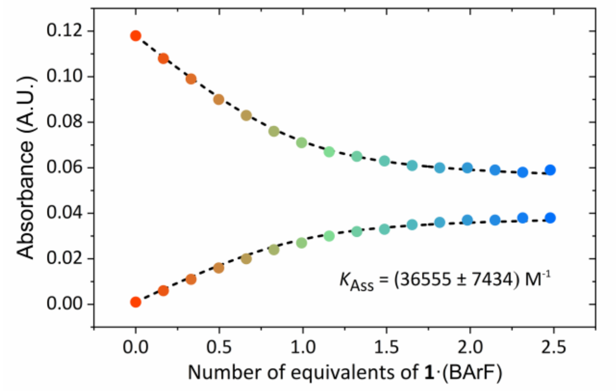

Figure S13 UV-Vis titration curve of the addition of **2** (0.5 mM) into a solution of **DCA** (0.2 mM) in CH_2Cl_2. The top and bottom curves are associated with the absorbance at 424 nm and 449 nm, respectively. The solid points represent the experimental data with the continuous dashed line represents the best-fit binding isotherm.

S4.3.4 Benzoquinone with **2**

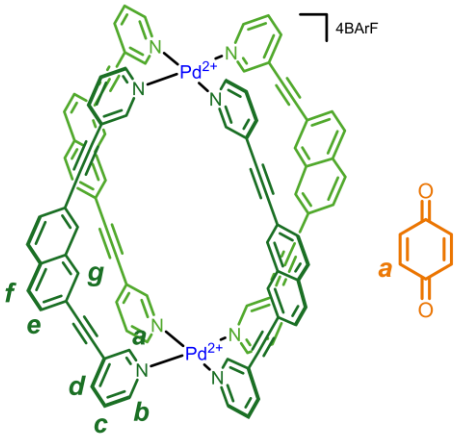

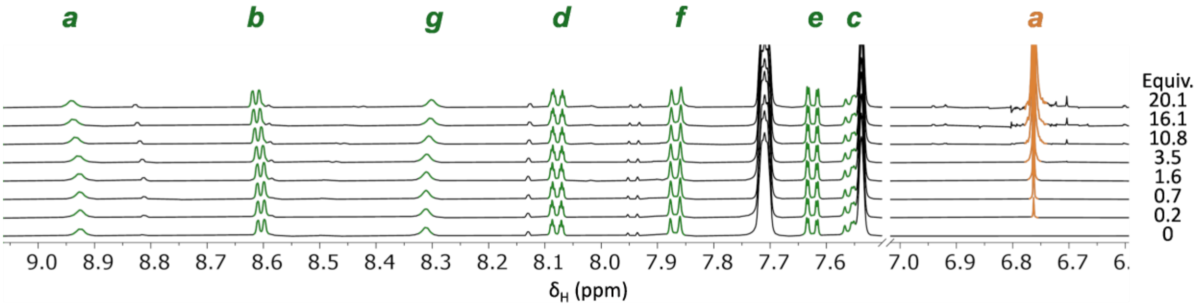

Figure S14 Partial ^{1}H NMR spectra (400 MHz, CD_2Cl_2, 300 K) of the titration of **2** (0.50 mM) with benzoquinone (25 mM).

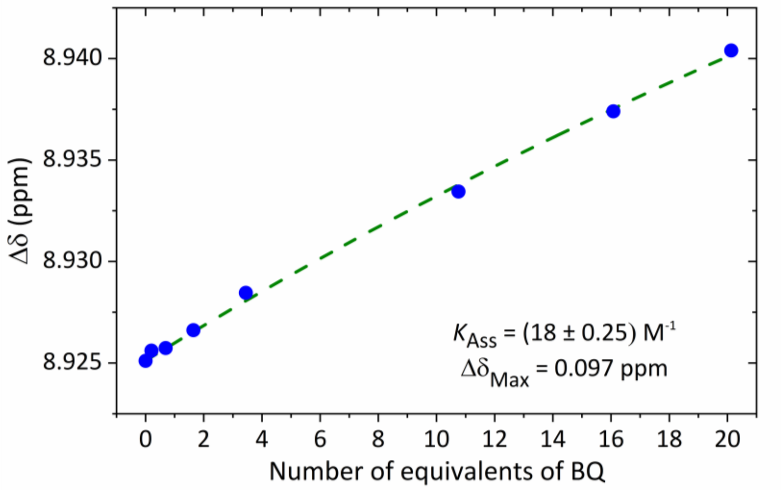

Figure S15 ^{1}H NMR (400 MHz, CD_2Cl_2, 300 K) titration curve of **2** (0.5 mM) with benzoquinone (25 mM). The curve was obtained by monitoring the internal cage cavity proton Ha. The solid points represent the experimental data with the continuous dashed line represents the best-fit binding isotherm.

Table S1 Comparison of the association constants (K_{Ass}) of quinone and dicyanoarene guests in **1** and **2**, respectively. All K_{Ass} were obtained in CD_2Cl_2 with BArF as the counterion.

**Guest⊂1**[S7]	K_{Ass} **(M^{-1})**	**Guest⊂2**	K_{Ass} **(M^{-1})**
Benzoquinone	7.24 ± 0.10 x 10^3	Dicyanobenzene	1.10 ± 0.04 x 10^3
Naphthoquinone	3.49 ± 0.47 x 10^5	Dicyanonaphthalene	2.14 ± 0.10 x 10^4
Anthraquinone	4.89 ± 0.38 x 10^7	Dicyanoanthracene	3.66 ± 0.74 x 10^4
		Benzoquinone	1.8 ± 0.03 x 10^1

S5. X-ray Crystallography

S5.1 General experimental details

Crystals were mounted on a MITIGEN holder in Paratone or perfluoroether oil on a Rigaku Oxford Diffraction SuperNova diffractometer and were kept at a steady at T = 120 K during data collection. The structures were solved with the ShelXT 2018/2 solution program using the Intrinsic Phasing solution method and by using Olex2 as the graphical interface.[S8,S9] The models were refined with ShelXL 2018/3 using full matrix least squares minimization on F^2.[S10]

S5.2 Crystallographic data and special refine details

S5.2.1 L

Crystal Data for L. Colorless needle-shaped crystal with dimensions 0.26 × 0.04 × 0.02 mm^3. $C_{24}H_{14}N_2$, M_r = 330.37, orthorhombic, a = 7.3310(4) Å, b = 39.373(2) Å, c = 5.7483(3) Å, α = 90°, β = 90°, γ = 90°, V = 1659.23(16) Å^3, Z = 4, $Pnma$, D_c = 1.323 g cm^{-3}, μ = 0.606 mm^{-1}, T = 120.01(10) K, 33414 reflections measured, 1487 unique (R_{int} = 0.1618) which were used in all calculations, wR_2 (all data) = 0.1925, and R_1 [I > 2(I)]= 0.0683. CCDC 2157965.

The value of Z' is 0.5. This means that only half of the formula unit is present in the asymmetric unit, with the other half consisting of symmetry equivalent atoms.

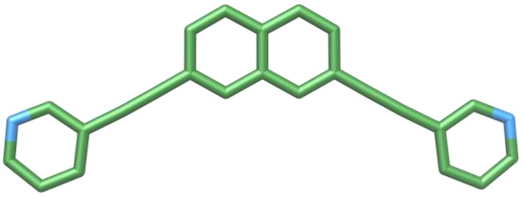

Figure S16 X-ray crystal structure of L. Protons have been removed for clarity. Color code: C: green, N: light blue.

S5.2.2 **2**

Crystal Data for 2. Colorless lath-shaped crystal with dimensions 0.35 × 0.04 × 0.02 mm^3. $C_{240}H_{144}B_4F_{96}N_8O_4Pd_2$, M_r = 5283.66, monoclinic, a = 14.2988(2) Å, b = 31.0387(12) Å, c = 27.0751(8) Å, α = 90°, β = 102.329(2), γ = 90°, V = 11739.2(6) Å^3, Z = 2, $P2_1/c$, D_c = 1.495 g cm^{-3}, μ = 2.430 mm^{-1}, T = 120.0 K, 69156 reflections measured, 13879 unique (R_{int} = 0.0978) which were used in all calculations, wR_2 (all data) = 0.2156, and R_1 [I > 2(I)]= 0.0748. CCDC 2157967.

Some of the -CF_3 groups were modelled as disordered, with appropriate similarity restraints. The resolution of the data set was cut at 0.96 Å, consistent with rapidly rising values of R_{int} at higher resolution. The value of Z' is 0.5. This means that only half of the formula unit is present in the asymmetric unit, with the other half consisting of symmetry equivalent atoms.

S5.2.3 **DCB⊂2**

Crystal Data for DCB⊂2. Colorless block-shaped crystal with dimensions 0.33 × 0.24 × 0.18 mm^3. $C_{245}H_{108}B_4Cl_{26}D_{26}F_{96}N_{10}Pd_2$, M_r = 6245.51, triclinic, a = 21.9061(4) Å, b = 24.0847(4) Å, c = 28.8091(4) Å, α = 109.2510(10)°, β = 108.3080(10)°, γ = 101.2250(10)°, V = 12847.2(4) $Å^3$, Z = 2, P-1, D_c = 1.615 g cm^{-3}, μ = 4.740 mm^{-1}, T = 120.0 K, 219013 reflections measured, 100506 unique (R_{int} = 0.1025) which were used in all calculations, wR_2 (all data) = 0.3298, and R_1 [I > 2(I)]= 0.1199. CCDC 2157968.

Crystals of these cage-type compounds are very susceptible to rapid desolvation. In an attempt to prevent crystal decomposition, a small amount of solution was poured onto a shallow well microscope slide onto which a drop of Fomblin oil had been placed. Crystals were removed from the NMR tube, from which they had grown, and transferred into the microscope well solution before being pushed into the Fomblin oil. Crystal decomposition occurred as was evident in the diffraction pattern. The diffraction pattern was handled as a non-merohedral twin consistent with two clearly different domains being observed in a reciprocal lattice viewer. These actually correspond to a split, rather than twinned diffraction pattern with component 2 rotated by 3.1204° around [-0.21 0.93 -0.31] (reciprocal) or [-0.05 1.00 0.03] (direct). Different integration options were explored and the twinned integration, with production of an hklf5 format reflection file, gives the most acceptable refinement. The model has its limitations; it was not possible to optimize the ShelXL weighting scheme. A check with PLATON SQUEEZE (using a LIST 8-style FCF file) shows no overlooked additional solvent. The cage structures (there are two half-cages per asymmetric unit, plus two half-guests) were easily identified from solution and refine well, with no issues. Refinement of the $BArF^-$ ions was more problematic. Two can be modelled with essentially no restraints. The $BArF^-$ ions containing B401 and B501 were modelled with geometric similarity restraints relating to the $BArF^-$ ions containing B201. Additionally, these two anions plus the B301-containing $BArF^-$ ion were modelled with the RIGU restraint applied. Despite this, some -CF_3 groups exhibit large displacement ellipsoids, which cannot be controlled by classical disorder modelling. They are thus left as they are. They do appear to correlate with the disordered solvent, methylene chloride-d_2. There are 13 molecules of solvent in the asymmetric unit. Most are disordered to a certain degree, consistent with the rapid desolvation observed during crystal mounting. Geometric and RIGU restraints were used where applicable. One of the methylene carbon atoms was refined using an isotropic model. In some instances, the methylene chloride-d_2 molecules have a close approach to -CF_3 groups, which also exhibit large ellipsoids. The apparent disorder is obviously correlated but could not be adequately modelled. There is a single molecule in the asymmetric unit, which is represented by the reported sum formula. In other words: Z is 2 and Z' is 1.

S5.2.4 **DCN⊂2**

Crystal Data for DCN⊂2. Colorless plate-shaped crystal with dimensions 0.32 × 0.27 × 0.04 mm^3.

$C_{244}H_{126}B_4Cl_{16}F_{96}N_{10}Pd_2$, M_r = 5844.78, monoclinic, a = 16.7163(2) Å, b = 19.2517(2) Å, c = 43.1012(4) Å, α = 90°, β = 96.5080(10)°, γ = 90°, V = 13781.3(3) $Å^3$, Z = 2, $P2_1/n$, D_c = 1.408 g cm^{-3}, μ = 3.511 mm^{-1}, T = 120.0 K, 210576 reflections measured, 28537 unique (R_{int} = 0.1122) which were used in all calculations, wR_2 (all data) = 0.3161, and R_1 [I > 2(I)]= 0.1100. CCDC 2157969.

The solvent masking routine of Olex2 was used to account for electron density relating to dichloromethane molecules which could not be modelled using discrete atoms. The value of Z' is 0.5. This means that only half of the formula unit is present in the asymmetric unit, with the other half consisting of symmetry equivalent atoms.

S5.2.5 **DCA⊂2**

Crystal Data for DCA⊂2. Fluorescent yellow plate-shaped crystal with dimensions 0.18 × 0.06 × 0.02 mm^3. $C_{258}H_{148}B_4Cl_{36}F_{96}N_{10}Pd_2$, M_r = 6744.10, monoclinic, a = 16.7569(8) Å, b = 19.2513(8) Å, c = 43.240(3) Å, α = 90°, β = 97.374(5)°, γ = 90°, V = 13833.3(13) Å^3, Z = 2, $P2_1/n$, D_c = 1.619 g cm^{-3}, μ = 5.321 mm^{-1}, T = 120.00(10) K, 22614 reflections measured, 5262 unique (R_{int} = 0.1124) which were used in all calculations, wR_2 (all data) = 0.2284, and R_1 [I > 2(I)]= 0.0827. CCDC 2157966.

The structure has been refined as far as is practical given the quality of the experimental data. ShelX-specific constraints and restraints are elaborated upon in the embedded .res file. Some particular details are: (1) The data resolution was cut at 1.4 Å. R_{merge} and I/σ(I) become too high and too low respectively at higher resolutions. This is still enough to identify all non-H atoms in the cage, guest and the counterions, plus some solvent molecules. (2) The solvent masking routine of Olex2 was used to handle four molecules of dichloromethane which can be identified from a difference map, but which do not refine well. (3) All B, C and N atoms, plus some N atoms, were refined using an isotropic model as a significant proportion became non-positive definite when modelled anisotropically. (4) Distance similarity restraints were used on disordered -CF_3 groups and on the Pd-N distances. The value of Z' is 0.5. This means that only half of the formula unit is present in the asymmetric unit, with the other half consisting of symmetry equivalent atoms.

S5.2.6 **TCDCB⊂2**

Crystal Data for TCDCB⊂2. Yellow prism-shaped crystal with dimensions 0.29 × 0.19 × 0.13 mm^3. $C_{244}H_{56}B_4Cl_{28}D_{24}F_{96}N_{10}Pd_2$, M_r = 6247.96, monoclinic, a = 16.6784(3) Å, b = 19.2080(3) Å, c = 43.1505(5) Å, α = 90°, β = 97.0510(10)°, γ = 90°, V = 13719.1(4) Å^3, Z = 2, $P2_1/n$, D_c = 1.512 g cm^{-3}, μ = 4.619 mm^{-1}, T = 120.0 K, 215533 reflections measured, 25115 unique (R_{int} = 0.1181) which were used in all calculations, wR_2 (all data) = 0.3223, and R_1 [I > 2(I)]= 0.1242. CCDC 2157970.

The crystal was observed to desolvate on removal from the mother liquor. While the cage, guest and counterions all are reasonably well defined, the unit cell also contains a lot of dichloromethane solvent molecules. Much of this could be identified from successive difference Fourier maps and included in the model but electron density pertaining to 2.5 molecules per asymmetric unit - 10 per unit cell - could not, and so were removed with the SQUEEZE routine of PLATON. This triggers checkCIF alerts, which should be ignored. Some of the dichloromethane molecules were refined using an isotropic model as an anisotropic model proved to be unstable. Similarly, some of the -CF_3 groups which display large displacement ellipsoids have been modelled using restraints - RIGU in ShelXL - as disorder models were not stable. It was not possible to optimize the ShelXL weighting scheme, thus parameters which depend on this (*wR2*, GooF) could not be optimized. There is a single molecule in the asymmetric unit, which is represented by the reported sum formula. In other words: *Z* is 2 and *Z'* is 0.5.

S5.3 Crystallographic packing

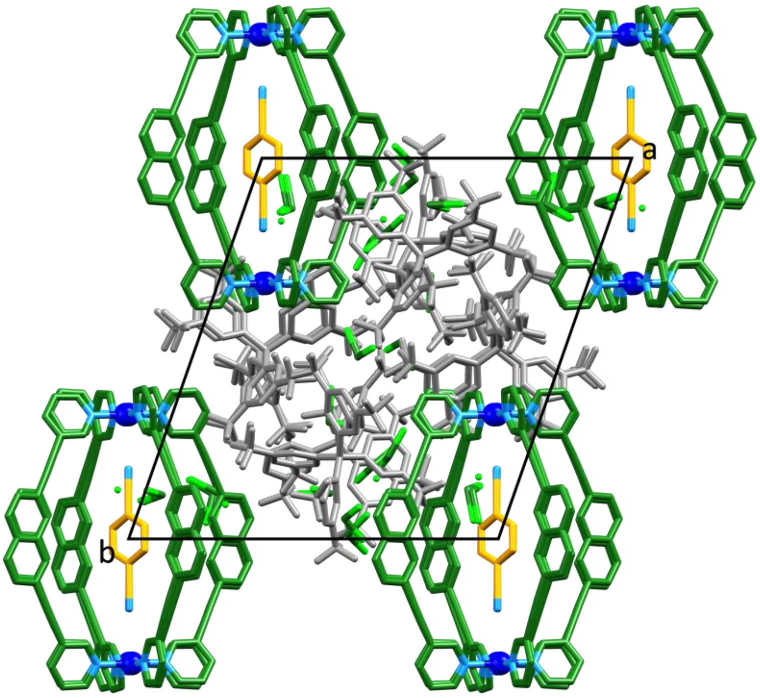

Figure S17 The crystal packing of **DCB⊂2** as viewed along the *c*-axis. The BArF⁻ ions are colored grey and the protons have been removed for clarity. Color code: C: green (host) or orange (guest), N: light blue, Pd: blue, Cl: light green.

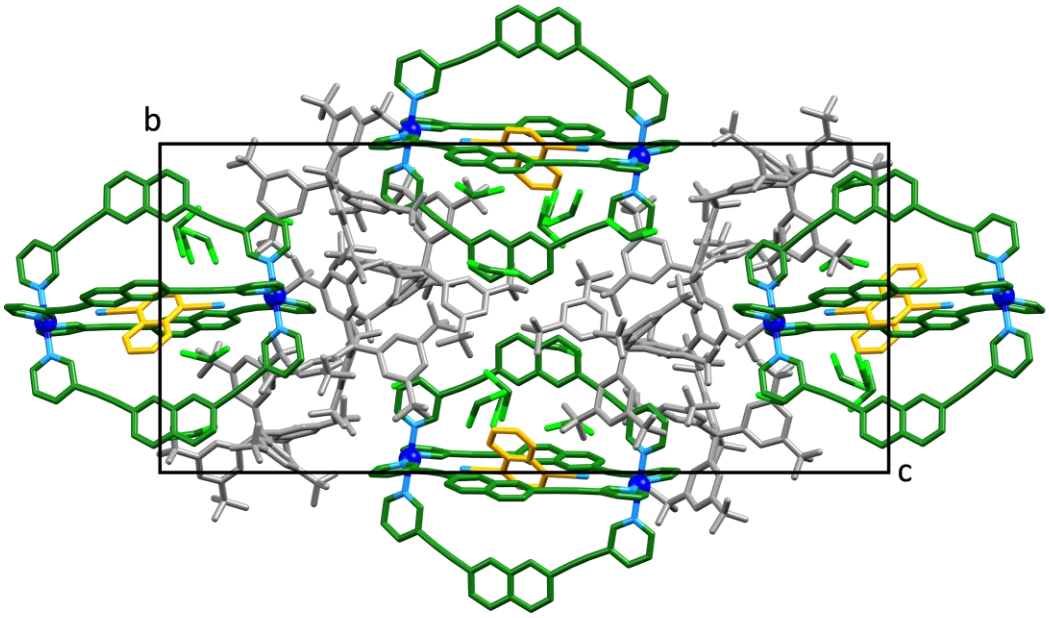

Figure S18 The crystal packing of **DCN⊂2** as viewed along the *a*-axis. The BArF⁻ ions are colored grey and the protons have been removed for clarity. Color code: C: green (host) or orange (guest), N: light blue, Pd: blue, Cl: light green.

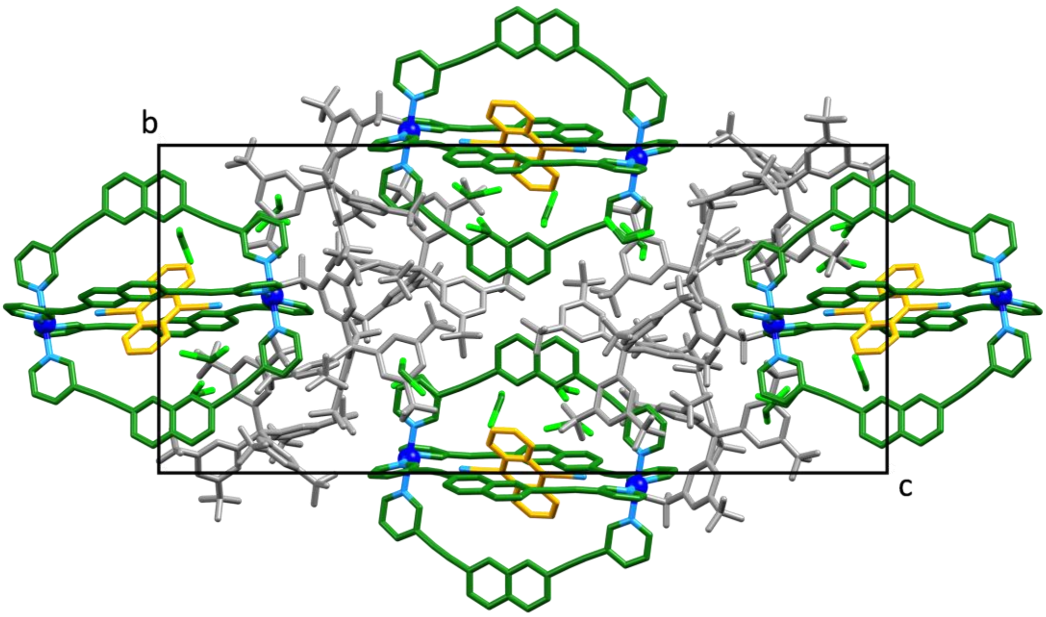

Figure S19 The crystal packing of **DCA⊂2** as viewed along the *a*-axis. The BArF⁻ ions are colored grey and the protons have been removed for clarity. Color code: C: green (host) or orange (guest), N: light blue, Pd: blue, Cl: light green.

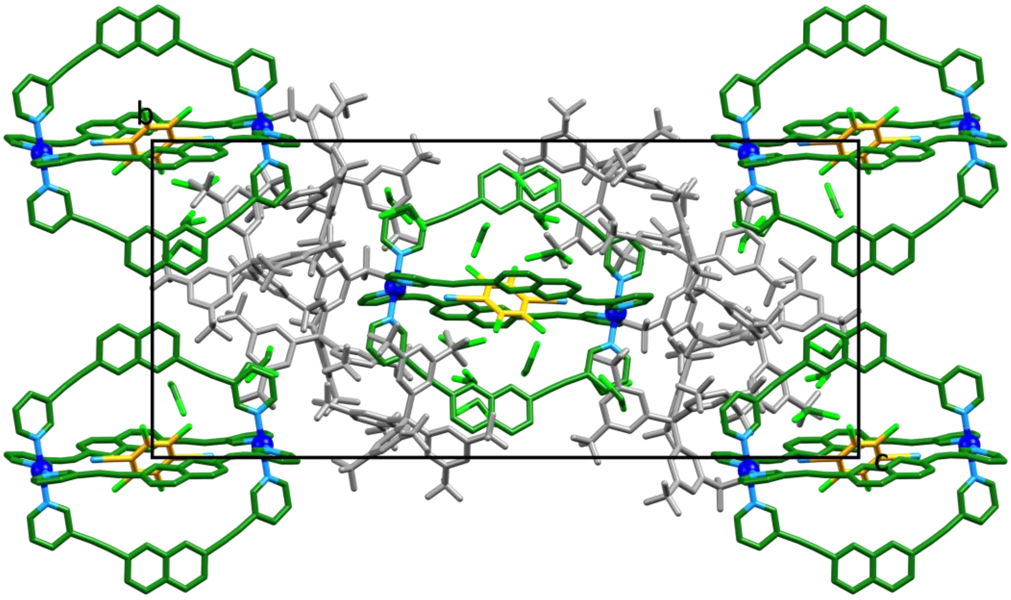

Figure S20 The crystal packing of **TCDCB⊂2** as viewed along the *a*-axis. The BArF⁻ ions are colored grey and the protons have been removed for clarity. Color code: C: green (host) or orange (guest), N: light blue, Pd: blue, Cl: light green.

S6. Raman Spectroscopy

Raman spectra were acquired on a Renishaw InVia Raman microscope equipped a 785 nm diode laser providing a maximum power of 300 mW using a 1200 l/mm grating. According to the Manufacturer's specification, the InVia Raman microscope has a spectral resolution of 0.5 cm^{-1} and high spectral stability to monitor minute shifts in Raman band position (as low as 0.02 cm^{-1}). For the solid-state studies, crystals of **2** and all host-guest complexes were collected and dried, before a small amount of solid was transferred onto a CaF_2 window and Raman spectra were acquired using λ_{ex} = 785 nm and a 5× NA 0.12 NPlanEPI objective (Leica), a 20× NA 0.4 NPlanEPI objective or a 50× NA 0.75 NPlanEPI objective (Leica). For the solution-state studies, solutions were prepared in dichloromethane (DCM) in a quartz cuvette (up to 500 µL) in a similar manner to that as described in Section S4.2, and Raman spectra were acquired using a 20× NA 0.4 NPlanEPI objective (Leica) using a 10 s acquisition time.

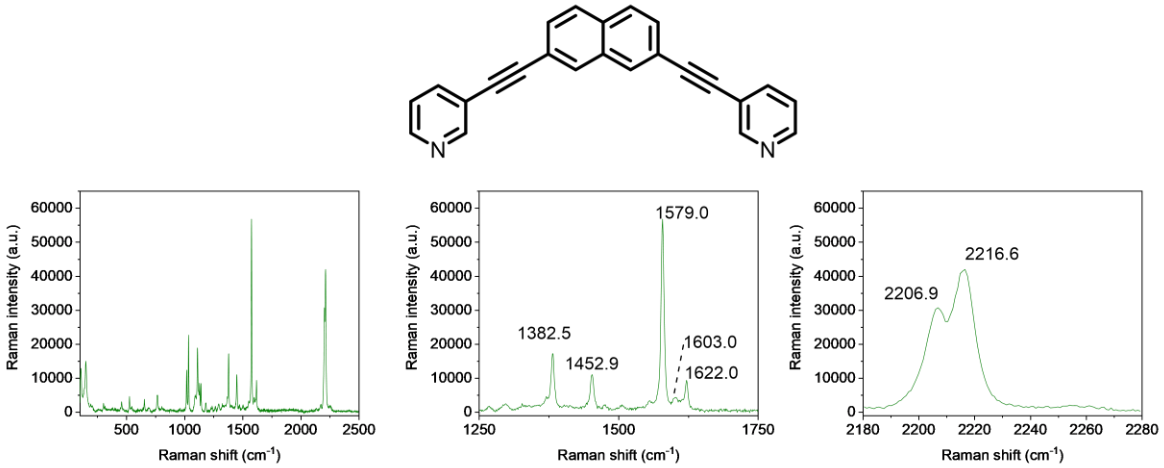

Figure S21 Raman spectrum of L in solid-state. Raman spectra were acquired using 785 nm excitation for 10 s using a 50× objective lens (0.18 mW). All assignments are in cm^{-1}.

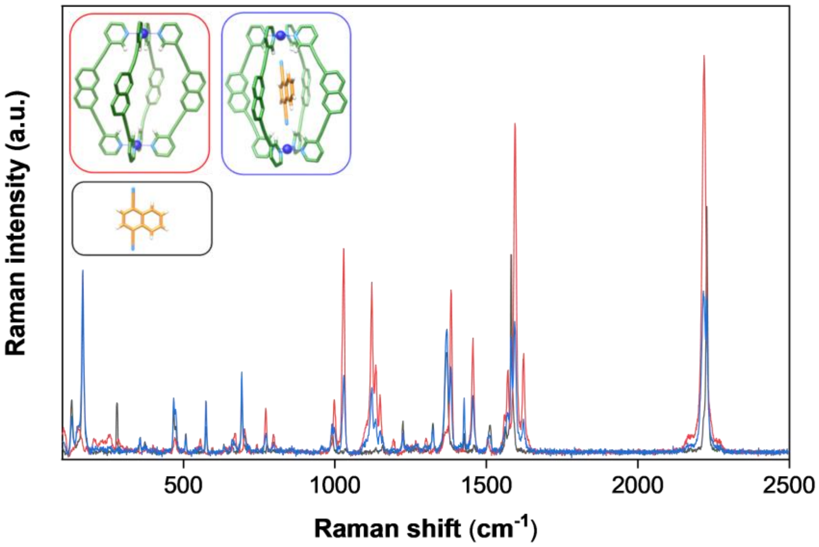

Figure S22 Solid-state analysis of dicyanonaphthalene (**DCN**) encapsulation using Raman spectroscopy. Raman spectra were acquired from the unbound guest (**DCN**; black) and cage (**2**; red) and the host-guest complex (**DCN⊂2**; blue). Raman spectra were acquired using 785 nm excitation for 10 s using a 50× objective lens (0.18 mW). Peak annotations are in cm^{-1}.

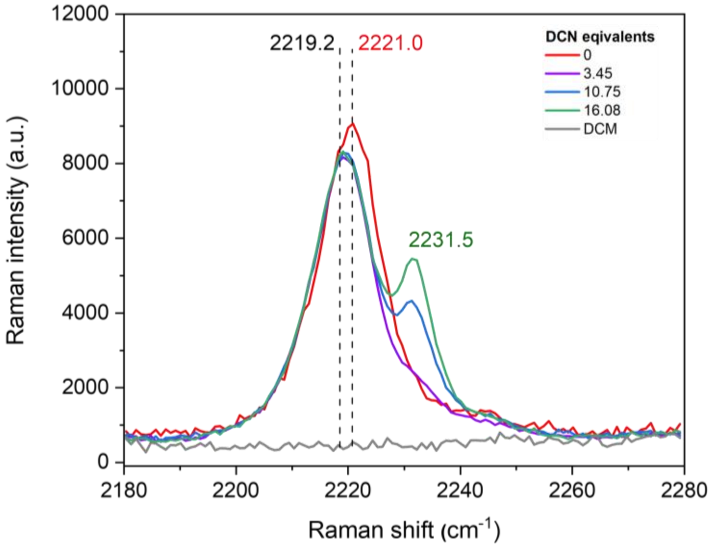

Figure S23 Solution-state analysis of dicyanonaphthalene (**DCN**) encapsulation using Raman spectroscopy. Raman spectra were acquired from the titration of **2** (0.50 mM) with **DCN** (25 mM) in dichloromethane (DCM). Raman spectra were acquired using 785 nm excitation for 20 s with a 5× objective lens (~180 mW). Peak annotations are in cm^{-1}.

Table S2 Summary of key Raman bands in the solid-state spectra of the host-guest complexes.

Guest	ν(ring); naph.[S11]	ν(ring); naph.[S11]	ν(ring); pyr.[S12] & naph.[S11]	ν(CC); pyr.[S12]	ν(ring); naph.[S11]	ν(C≡C); lantern[a]
-	1385.7	1458.2	1574.1	1597.2	1626.1	2222.0
**DCB**	1383.7	1458.2	1574.1	1597.2	1624.1	2222.0
**DCN**	1383.7	1458.2	1573.1	1596.2	1625.1	2220.2
**DCA**	1384.7	1458.2	1575.1	1598.2	1623.1	2220.0
**TCDCB**	1384.7	1457.2	1573.1	1595.2	1622.1	2222.9; 2213.2

[a]The peaks reported here are the most intense at that region. In some cases, the ν(C≡N) may contribute to the peak shape and spectral position.

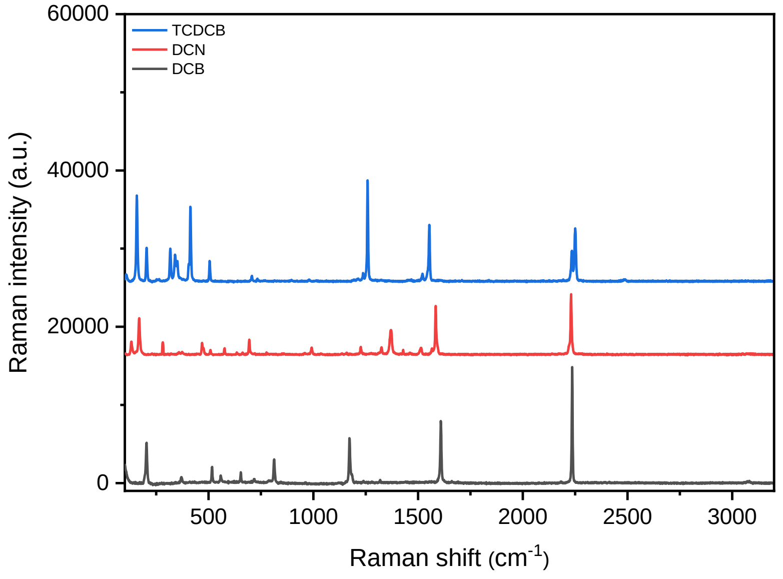

Figure S24 Raman spectral analysis of the free guests. Raman spectra were acquired in solid-state using 785 nm excitation for 10 s with a 50× objective lens (~0.18 mW). **DCB** (dicyanobenzene, bottom, black line), **DCN** (dicyanonaphthalene, middle, red line), **TCDCB** (tetrachlorodicyanobenzene, top, blue line). A complete Raman spectrum of **DCA** (dicyanoanthracene) could not be acquired due to the fluorescent nature of this guest at 532, 633 and 785 nm. A partial Raman spectrum indicated detection of the nitrile band at 2220.8 cm^{-1} (see Figure S25).

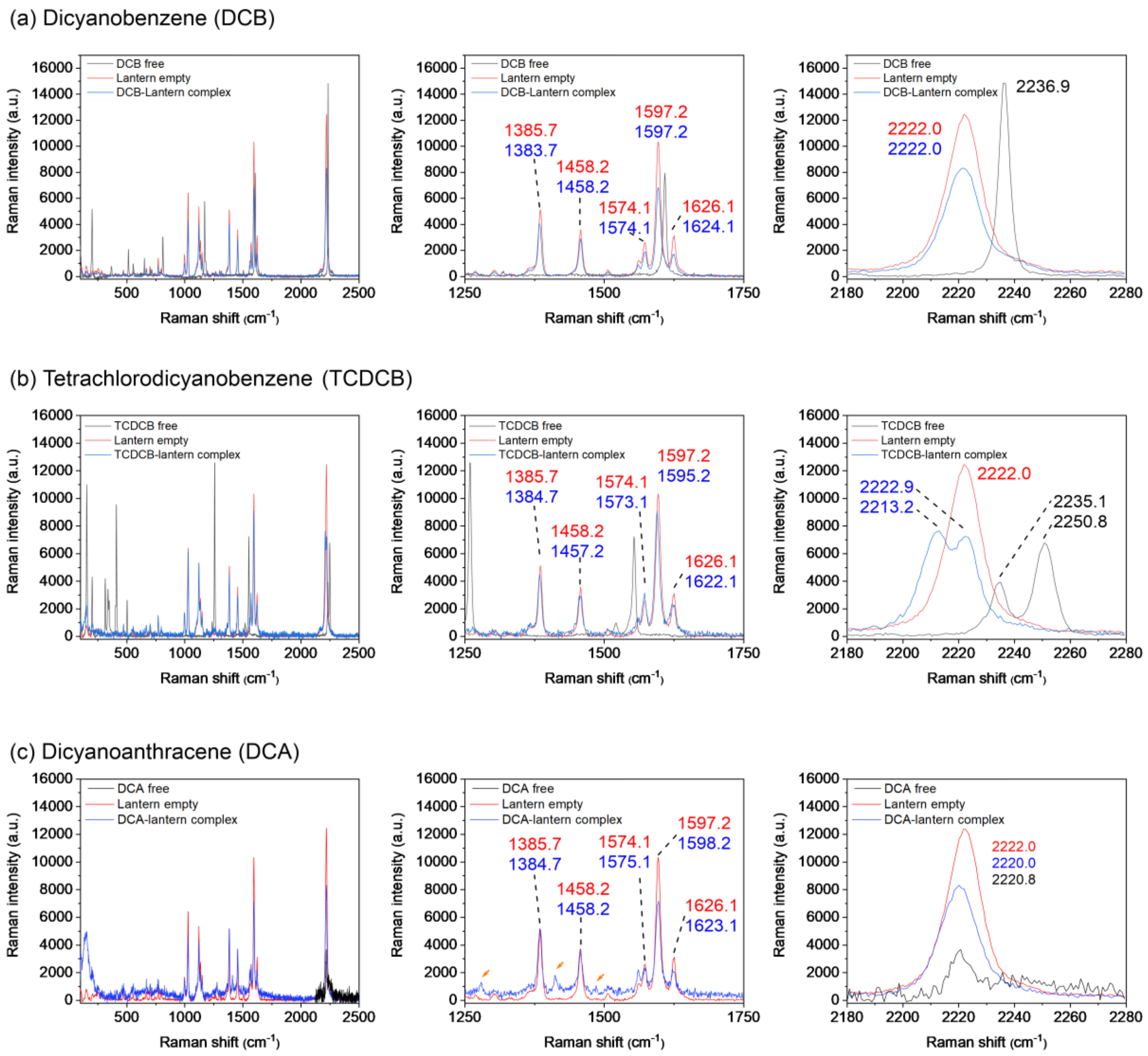

Figure S25 Analysis of guest encapsulation in the solid-state using Raman spectroscopy. Raman spectra were acquired from the free guest (black), the free lantern, **2** (red), and the host-guest complex (blue). Raman spectra were acquired using 785 nm excitation for 10 s using a 50× objective lens (0.18 mW). Raman spectra are presented in the range 100–2500 cm^{-1} (left), expanded view from 1250–1750cm^{-1} (center) and expanded view 2180–2280 cm^{-1} (right). Analysis of (a) dicyanobenzene (**DCB**), (b) tetrachlorodicyanobenzene (**TCDCB**), and (c) dicyanoanthracene (**DCA**). The Raman spectrum for **DCA** (free) is presented between 2100–2500 cm^{-1} only due to the broad fluorescent background associated with this compound. In addition, arrowheads are added to indicate Raman peaks likely to arise from the guest molecule. All assignments are color coded to the relevant spectrum and are presented in cm^{-1}.

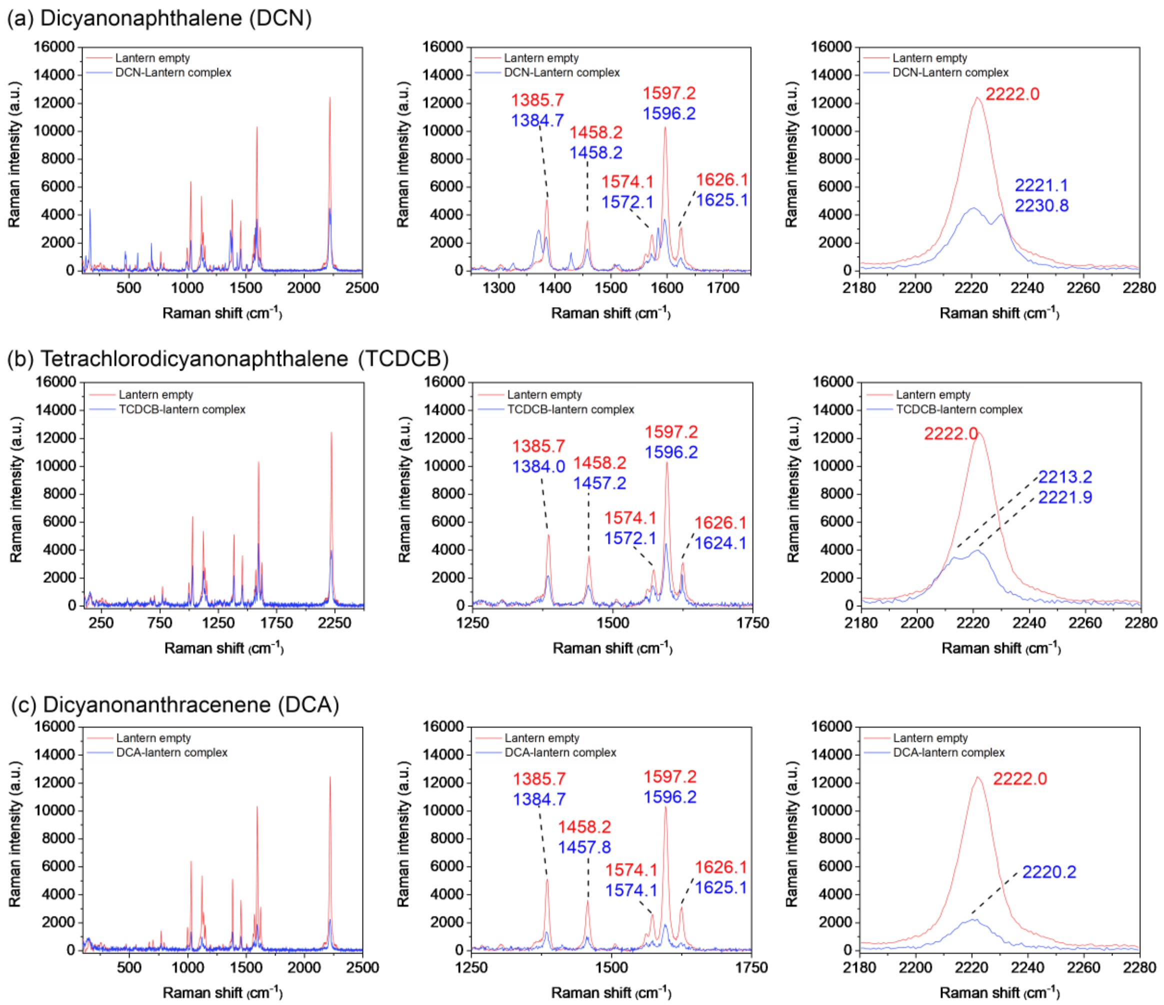

Figure S26 Repeat analysis of guest encapsulation in the solid-state using Raman spectroscopy. Raman spectra were acquired the free lantern, **2** (red), and the host-guest complex (blue). Raman spectra were acquired using 785 nm excitation for 10 s using a 50× objective lens (0.18 mW). Raman spectra are presented in the range 100–2500 cm^{-1} (left), expanded view from 1250–1750cm^{-1} (center) and expanded view 2180–2280 cm^{-1} (right). Analysis of (a) dicyanonaphthalene (**DCN**), (b) tetrachlorodicyanobenzene (**TCDCB**), and (c) dicyanoanthracene (**DCA**). All assignments are color coded to the relevant spectrum and are presented in cm^{-1}.

S7. References

S1. Kilpin, K. J.; Gower, M. L.; Telfer, S. G.; Jameson, G. B.; Crowley, J. D. Toward the Self-Assembly of Metal-Organic Nanotubes Using Metal-Metal and π-Stacking Interactions: Bis (Pyridylethynyl) Silver(I) Metallo-Macrocycles and Coordination Polymers. *Inorg. Chem.* **2011**, *50* (3), 1123−1134. https://doi.org/10.1021/ic1020059.

S2. *Spectral Database for Organic Compounds (SDBS)*. National Institute of Advanced Industrial Science and Technology. ^{1}H NMR; SDBS No.: 2378; CAS Registry Number 623-26-7. https://sdbs.db.aist.go.jp (accessed 2021-04-05).

S3. Gopi, E.; Gravel, E.; Doris, E. Triphenylbismuth Dichloride-Mediated Conversion of Thioamides to Nitriles. *Eur. J. Org. Chem.* **2019**, *2019* (25), 4043−4045. https://doi.org/10.1002/ejoc.201900563.

S4. Glöcklhofer, F.; Lunzer, M.; Stöger, B.; Fröhlich, J. A Versatile One-Pot Access to Cyanoarenes from ortho- and para-Quinones: Paving the Way for Cyanated Functional Materials. *Chem. Eur. J.* **2016**, *22* (15), 5173−5180. https://doi.org/10.1002/chem.201600004.

S5. Strohalm, M.; Kavan, D.; Novák, P.; Volný, M.; Havlíček, V. mMass 3: A Cross-Platform Software Environment for Precise Analysis of Mass Spectrometric Data. *Anal. Chem.* **2010**, *82* (11), 4648–4651. https://doi.org/10.1021/ac100818g.

S6. Martí-Centelles, V.; Lawrence, A. L.; Lusby, P. J. High Activity and Efficient Turnover by a Simple, Self-Assembled "Artificial Diels–Alderase". *J. Am. Chem. Soc.* **2018**, *140* (8), 2862−2868. https://doi.org/10.1021/jacs.7b12146.

S7. August, D. P.; Nichol, G. S.; Lusby, P. J. Maximizing Coordination Capsule–Guest Polar Interactions in Apolar Solvents Reveals Significant Binding. *Angew. Chem. Int. Ed.* **2016**, *55* (48), 15022−15026. https://doi.org/10.1002/anie.201608229.

S8. Sheldrick, G. M. SHELXT - Integrated space-group and crystal-structure determination. *Acta Crystallogr., Sect. A: Found. Adv.* **2015**, *71*, 3−8, https://doi.org/10.1107/S2053273314026370.

S9. Dolomanov, O. V.; Bourhis, L. J.; Gildea, R. J.; Howard, J. A. K.; Puschmann, H. OLEX2: a complete structure solution, refinement and analysis program. *J. Appl. Cryst.* **2009**, *42*, 339−341. https://doi.org/10.1107/S0021889808042726.

S10. Sheldrick, G. M. Crystal structure refinement with SHELXL. *Acta Crystallogr., Sect. C: Struct. Chem.* **2015**, *71*, 3−8. https://doi.org/10.1107/S2053229614024218.

S11. Lokshin, B. V.; Borisova, N. E.; Senyavin, B. M.; Reshetova, M. D. Analysis of vibrational spectra of naphthalene, deuterionaphthalenes, and chromium (η6-naphthalene)tricarbonyl based on density functional calculations. *Russ. Chem. Bull. Int. Ed.* **2002**, *51* (9), 1656−1666. https://doi.org/10.1023/A:1021339102661.

S12. Partal Ureña, F.; Fernández Gómez, M.; López González, J. J.; Martínez Torres, E. A new insight into the vibrational analysis of pyridine. *Spectrochim. Acta, Part A,* **2003**, *59* (12), 2815−2839. https://doi.org/10.1016/S1386-1425(03)00082-9.
